# The mammalian peroxisomal membrane is permeable to both GSH and GSSG – Implications for intraperoxisomal redox homeostasis

**DOI:** 10.1016/j.redox.2023.102764

**Published:** 2023-05-25

**Authors:** Maria J. Ferreira, Tony A. Rodrigues, Ana G. Pedrosa, Luís Gales, Armindo Salvador, Tânia Francisco, Jorge E. Azevedo

**Affiliations:** a Instituto de Investigação e Inovação em Saúde (I3S), Universidade do Porto, Rua Alfredo Allen, 208, 4200-135, Porto, Portugal; b Instituto de Biologia Molecular e Celular (IBMC), Universidade do Porto, Rua Alfredo Allen, 208, 4200-135, Porto, Portugal; c Instituto de Ciências Biomédicas de Abel Salazar (ICBAS), Universidade do Porto, Rua de Jorge Viterbo Ferreira, 228, 4050-313, Porto, Portugal; d Coimbra Chemistry Center—Institute of Molecular Sciences (CQC-IMS), University of Coimbra, 3004-535, Coimbra, Portugal; e CNC—Center for Neuroscience and Cell Biology, 3004-504, Coimbra, Portugal; f Institute for Interdisciplinary Research, University of Coimbra, 3030-789, Coimbra, Portugal

**Keywords:** Peroxisome, Glutathione, Membrane permeability, Glutaredoxin, Protein import, Kinetic simulation

## Abstract

Despite the large amounts of H_2_O_2_ generated in mammalian peroxisomes, cysteine residues of intraperoxisomal proteins are maintained in a reduced state. The biochemistry behind this phenomenon remains unexplored, and simple questions such as “is the peroxisomal membrane permeable to glutathione?” or “is there a thiol-disulfide oxidoreductase in the organelle matrix?” still have no answer. We used a cell-free *in vitro* system to equip rat liver peroxisomes with a glutathione redox sensor. The organelles were then incubated with glutathione solutions of different redox potentials and the oxidation/reduction kinetics of the redox sensor was monitored. The data suggest that the mammalian peroxisomal membrane is promptly permeable to both reduced and oxidized glutathione. No evidence for the presence of a robust thiol-disulfide oxidoreductase in the peroxisomal matrix could be found. Also, prolonged incubation of organelle suspensions with glutaredoxin 1 did not result in the internalization of the enzyme. To explore a potential role of glutathione in intraperoxisomal redox homeostasis we performed kinetic simulations. The results suggest that even in the absence of a glutaredoxin, glutathione is more important in protecting cysteine residues of matrix proteins from oxidation by H_2_O_2_ than peroxisomal catalase itself.

## Highlights

• The mammalian peroxisomal membrane is permeable to GSH and GSSG.
• No robust glutaredoxin activity was found in peroxisomes.
• Kinetic simulations suggest that GSH protects proteins from H_2_O_2_ even in the absence of glutaredoxin.
• The protective effect of GSH is larger than that of catalase.

## Graphical abstract

**Figure:**
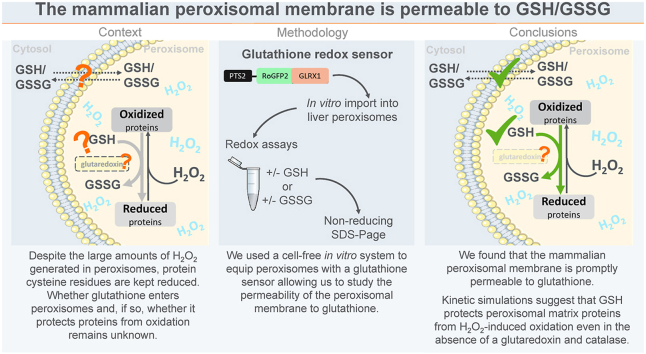

## 1 Introduction

Peroxisomes contain many H_2_O_2_-producing oxidases in their lumen [1]. In mammals, these oxidases are involved in several metabolic pathways such as fatty acid beta-oxidation, bile acid synthesis and purine and d-amino acid catabolism [2]. Together, they may consume up to 20% of the O_2_ that enters the liver, thus generating large amounts of H_2_O_2_ [1]. Hydrogen peroxide is a relatively stable oxidant but, nevertheless, it can oxidize organic molecules directly [3]. Also, in the presence of transition metal ions, H_2_O_2_ can undergo Fenton chemistry yielding much more reactive species such as the hydroxyl radical [4].

To neutralize H_2_O_2_ and other reactive species, peroxisomes are equipped with a small set of antioxidant enzymes (reviewed in Refs. [5,6]). By far the most abundant is catalase, an enzyme that comprises ∼15% of the total protein in liver peroxisomes and that dismutates H_2_O_2_ into O_2_ and water [7]. In addition to catalase, proteomics analyses of highly pure peroxisomes [[8], [9], [10]] have shown that these organelles also contain small fractions of superoxide dismutase 1 [11], epoxide hydrolase 2 [12], peroxiredoxin 5 (PRDX5 [13]) and glutathione S-transferase kappa 1 (GSTk1 [14]), four enzymes that display multiple subcellular localizations.

The biological roles of catalase, superoxide dismutase 1 and epoxide hydrolase 2 are relatively well established [7,12,15]. However, there are still some questions regarding the exact roles of PRDX5 and GSTk1 in the peroxisome. Specifically, it is unclear how PRDX5, a peroxiredoxin that undergoes oxidation/reduction cycles during detoxification of hydroperoxides [16], is reduced in the peroxisomal lumen since it is likely that there is no thioredoxin system in peroxisomes (see below). Regarding GSTk1, it is known that the enzyme protects cells from reactive oxygen species (ROS)-induced cell death [17] and that it displays some glutathione transferase and glutathione peroxidase activities in in *vitro* assays with a few substrates [14,18]. However, the type of reaction catalyzed by GSTk1 *in vivo* remains unknown. Indeed, GSTk1 is structurally more similar to bacterial 2-Hydroxychromene-2-carboxylic acid Isomerases than to canonical/cytosolic GSTs [19,20]. Those bacterial members of the GST kappa family also display glutathione transferase activity in *in vitro* assays; however, their function is to catalyze a *cis-trans* isomerization within the naphthalene catabolic pathway, a reaction in which GSH is used as a cofactor and not as a substrate (*i.e.*, GSH is not consumed [20,21]).

A striking property of the five enzymes mentioned above is that the complete absence of any of them in mice has no implications to peroxisome function [[22], [23], [24], [25], [26], [27]]. Catalase knockout mice, for example, do not present any morphological/biochemical phenotypes associated with peroxisome defects [23] and, in fact, develop “normally and were apparently healthy upon observation up to 1 year of age" [27]. Importantly, analyses of cells from those mice using a peroxisome-targeted redox sensitive green fluorescent protein (roGFP2) revealed an unaltered thiol-disulfide redox potential of the peroxisomal matrix ([28]; see also below). Exactly how peroxisomal components escape massive oxidation in an organelle that lacks catalase remains enigmatic.

Protein cysteine residues are main targets of oxidation by H_2_O_2_ and other ROS, particularly when the pKa of their thiol groups is low, as it is the case for catalytic cysteine residues in many enzymes [3,29]. Oxidation of cysteine thiol group yields a variety of products (*e.g.*, sulfenic, disulfides, sulfinic and sulfonic derivatives) leading to protein dysfunction and enzyme inactivation [3]. Interestingly, despite the large amounts of H_2_O_2_ generated in peroxisomes, the thiol-disulfide redox potential of the peroxisomal matrix is similar to the one in the cytosol, as assessed with two cysteine-based redox probes, roGFP2 and Redoxfluor [28,30]. Although the redox pair(s) with which those probes equilibrate inside peroxisomes is still unknown (see Ref. [31] for a discussion of this issue), those findings do suggest that cysteine residues in peroxisomal matrix proteins are maintained as reduced as those of cytosolic proteins.

In other subcellular compartments, such as the cytosol and mitochondria, cysteine residues are maintained in the reduced state by the thioredoxin reductase-thioredoxin system and/or the glutathione reductase-glutathione-glutaredoxin system [32,33]. Despite intensive biochemical studies and several proteomic analyses of highly pure peroxisomes, neither thioredoxin reductase (TrxR) nor glutathione reductase were ever found in mammalian peroxisomes [[8], [9], [10],[34], [35], [36]]. Occasionally, a thioredoxin (Trx) or a glutaredoxin was identified in a proteomic analysis [9,10]. However, as trace amounts of several *bona fide* cytosolic/mitochondrial components are always identified in this type of studies, and neither the thioredoxin nor the glutaredoxin detected in purified peroxisomes have a peroxisomal targeting signal, it is unclear whether these proteins are true peroxisomal components or contaminants.

Given the absence of thioredoxin reductase in the peroxisome matrix, a thioredoxin-based system operating inside the organelle would require either the bidirectional transport of thioredoxin across the organelle membrane during each cystine reduction cycle, or a transmembrane protein with the capacity to convey reducing equivalents from the cytosol into the organelle matrix. However, it is unclear whether peroxisomes can export any of its matrix proteins [37] and, likewise, no evidence for a peroxisomal transmembrane protein displaying thiol-disulfide oxidoreductase activity was ever found. A glutaredoxin-based system, in contrast, would require fulfillment of two simple conditions: 1) the presence of a glutaredoxin in the organelle matrix, and 2) a membrane permeable to both reduced (GSH) and oxidized (GSSG) glutathione. As stated above it is presently unclear whether there is a glutaredoxin in the peroxisomal matrix. Regarding the permeability of the mammalian peroxisomal membrane to GSH/GSSG there are simply no data. We note that the presence of GSTk1 in the organelle matrix does not necessarily imply that the peroxisomal membrane is permeable to GSH. Indeed, if GSTk1 uses GSH as a cofactor and not as a substrate, the enzyme might simply acquire GSH in the cytosol, prior to import into the organelle, as proposed for some other co-factor containing peroxisomal enzymes [38].

Here we provide data suggesting that the mammalian peroxisomal membrane is, indeed, permeable to GSH and GSSG, and that the intraperoxisomal and cytosolic pools of glutathione are redox linked. No robust glutaredoxin activity was detected in peroxisomes. Unexpectedly, however, kinetic simulations suggest that glutathione plays a key role in maintaining intraperoxisomal redox homeostasis even in the absence of such an enzyme.

## 2 Results and discussion

### 2.1 Development of a cell-free *in vitro* system to study the permeability of the peroxisomal membrane to glutathione

A simple biochemical assay that has provided valuable information on the permeability of endoplasmic reticulum and mitochondrial membranes to glutathione consists of incubating those organelles in glutathione solutions of different redox potentials and determine whether the redox state of cysteine residues in intraorganellar proteins is altered [39,40]. We reasoned that such a strategy might also be of use to study peroxisomes and that a rat liver post-nuclear supernatant (PNS)-based system that we have been using to dissect the mechanism of protein import into peroxisomes [41] could be adapted for this purpose.

A first step towards this aim was to equip peroxisomes with a reporter protein that responds to glutathione. Fusion proteins comprising glutaredoxin and roGFP2 respond rapidly and specifically to glutathione and are widely used to measure the redox potential of the GSH/GSSG pair [42,43]. Specificity is imparted by the glutaredoxin domain, which transmits redox equivalents from the GSH/GSSG redox pair to two cysteines engineered in roGFP2. When the GSH/GSSG ratio is high, the two roGFP2 cysteines are in the reduced state whereas at lower GSH/GSSG ratios they form a disulfide bond. As oxidation of roGFP2 changes the spectral properties of its fluorophore, the redox state of the probe is frequently monitored fluorometrically [44]. Another way to quantify its redox state explores the fact that oxidized roGFP2 runs slightly faster than the reduced protein upon non-reducing SDS-PAGE, due to the presence of the disulfide bond [45,46]. In this work, we used the latter method.

We synthesized *in vitro* a radiolabeled chimeric protein comprising a cleavable peroxisomal targeting signal type 2 (PTS2), roGFP2 and glutaredoxin 1 (PTS2-roGFP2-GLRX1) and asked whether the protein could be imported into peroxisomes in the PNS-based system. For reasons that will become apparent later, a version of this protein lacking the glutaredoxin 1 (GLRX1) moiety (PTS2-roGFP2) was also synthesized and included in most experiments.

**Fig. 1:**
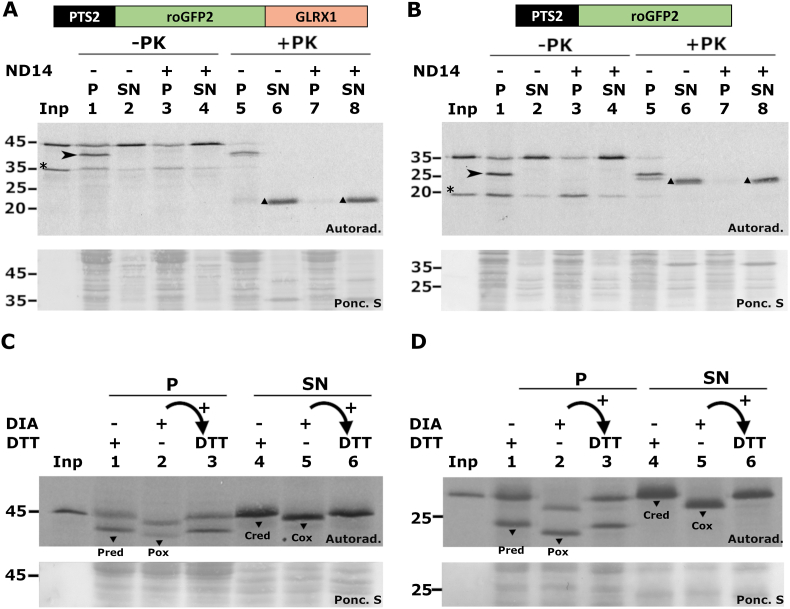
**– Cell-free peroxisomal *in vitro* import and redox properties of PTS2-roGFP2-GLRX1 and PTS2-roGFP2. A, B -** Radiolabeled PTS2-roGFP2-GLRX1 (A) and PTS2-roGFP2 (B) were subjected to *in vitro* import assays in the presence (+) or absence (−) of recombinant NDPEX14 (ND14). After import, the reactions were subjected (+PK) or not (-PK) to proteinase K treatment and centrifuged to separate organelles (“P”) from soluble (“SN”) proteins. Samples were analyzed by reducing SDS-PAGE/autoradiography. Arrow heads indicate the processed (intraperoxisomal) reporter proteins. The asterisks indicate proteins generated by autofragmentation of roGPF2 during chromophore rearrangement [106]. Note also that the GFP domain is intrinsically resistant to proteolysis [107] yielding a fragment upon proteinase K treatment (triangles). **C, D -** PNSs from *in vitro* import assays performed with either PTS2-roGFP2-GLRX1 (C) or PTS2-roGFP2 (D) were incubated for 5 min at 37 °C with 0.25 mM diamide (DIA) or 10 mM dithiothreitol (DTT), as indicated. After blocking cysteine residues with NEM, samples were centrifuged to separate organelles (“P”; lanes 1–3) from cytosolic proteins (“SN”; lanes 4–6). After TCA precipitation, proteins were solubilized in Laemmli buffer lacking reducing agents and containing NEM instead (lanes 1, 2, 4 and 5) and subjected to non-reducing SDS-PAGE/autoradiography. An aliquot of the diamide-treated samples was reduced with DTT and alkylated just before electrophoresis (lanes 3 and 6). Pred and Pox - reduced and oxidized peroxisomal reporter proteins, respectively. Cred and Cox - reduced and oxidized cytosolic reporter proteins, respectively. Autoradiographs (“Autorad.”) and the corresponding ponceau S-stained membranes (“Ponc. S”) are shown; lanes Inp, radiolabeled proteins used in the assays; numbers to the left indicate the molecular weight markers (in kDa).

Peroxisomal *in vitro* import of PTS2-containing proteins employing the PNS-based system is experimentally well characterized and can be assessed by several criteria [[47], [48], [49], [50]]. For instance, after incubation with the PNS, a fraction of the PTS2 protein should appear in the organelle pellet and should display a lower molecular mass, reflecting processing of the PTS2 peptide by the peroxisomal matrix TYSD1 protease [51]. This processed species should resist treatment of the PNS with an aggressive protease (*e.g.*, proteinase K [41]) because a protein that resides in the organelle lumen is protected from a soluble protease by the organelle membrane. Also, when the *in vitro* assay is done in the presence of a recombinant protein (referred to as NDPEX14 [41]) that binds and inactivates PEX5, the shuttling receptor that transports the PTS2 protein to the peroxisome, no processed protease-resistant radiolabeled protein should be detected in the organelle fraction. As shown in Fig. 1A, a fraction of the radiolabeled PTS2-roGFP2-GLRX1 (lane “Inp”) was converted to a smaller species upon incubation with the PNS (lane 1, arrowhead). This species is detected only in the organelle fraction (Fig. 1A, compare lanes 1 and 2) and is protease-protected (lane 5). Some intact/unprocessed 45-kDa protein can also be detected in the organelle fraction; however, the majority of this protein is simply adsorbed to the outer surface of the organelles because it is accessible to proteinase K (Fig. 1A, compare lanes 1 and 5). Importantly, no processed PTS2-roGFP2-GLRX1 is found in organelles when the *in vitro* assay is performed in the presence of NDPEX14 (Fig. 1A, lane 3) and, accordingly, no protease-protected species is detected under these conditions (lane 7), as expected. Thus, a fraction of the PTS2-roGFP2-GLRX1 protein is imported into peroxisomes in the PNS-based *in vitro* assay. The same results were obtained with the PTS2-roGFP2 protein (see Fig. 1B).

We next used a previously described strategy [45,46] to determine whether the two roGFP2 proteins present the expected redox properties. Specifically, we treated PNSs containing the reporter proteins with either diamide (a membrane permeable thiol-specific oxidant [52]) or dithiothreitol (DTT; a membrane-permeable reagent that reduces accessible disulfide bonds [53]). After blocking the remaining thiol groups with N-ethylmaleimide (NEM), samples were centrifuged to separate organelles from cytosolic proteins and analyzed by non-reducing SDS-PAGE/autoradiography. As shown in Fig. 1C, both the peroxisomal (processed) and cytosolic (unprocessed) populations of PTS2-roGFP2-GLRX1 display faster electrophoretic migrations in the diamide condition (lanes 2 and 5) than in the DTT condition (lanes 1 and 4). Also, when the proteins from the diamide-treated samples were reduced with DTT in the presence of SDS just before electrophoresis they recovered their slower migrations (Fig. 1C, lanes 3 and 6). Identical results were obtained with the PTS2-roGFP2 protein (Fig. 1D). We conclude that the roGFP2 moieties of the two reporter proteins are functional.

### 2.2 The peroxisomal membrane is promptly permeable to GSH and GSSG

To study the permeability of the peroxisomal membrane to GSH/GSSG, we developed a protocol comprising three steps: 1) the PNS is incubated with the radiolabeled reporter protein for 20 min to load peroxisomes with the redox sensor; 2) after stopping further import with NDPEX14 (2 min at 37 °C), the redox potential of the PNS is either left unchanged (under these conditions the reporter protein is mostly oxidized; see Materials and Methods for details) or is decreased by adding a small amount of tris(2-carboxyethyl)phosphine (TCEP), to reduce the reporter protein; 3) finally, a bolus of a reducing or oxidizing glutathione solution is added to the PNS and the reduction/oxidation kinetics of the reporter protein in both the soluble and organelle fractions are assessed by non-reducing SDS-PAGE. In addition to PTS2-roGFP2-GLRX1, PTS2-roGFP2 was also included in all the experiments described below, as a control. The two proteins can be reduced and oxidized by strong reductants and oxidants, respectively (*e.g.*, see Fig. 1B). However, only PTS2-roGFP2-GLRX1 responds rapidly to glutathione due to its GLRX1 domain, which accelerates the kinetics of redox equilibration between the roGFP2 moiety and the GSH/GSSG pair by a factor of at least 100 000 [42].

**Fig. 2:**
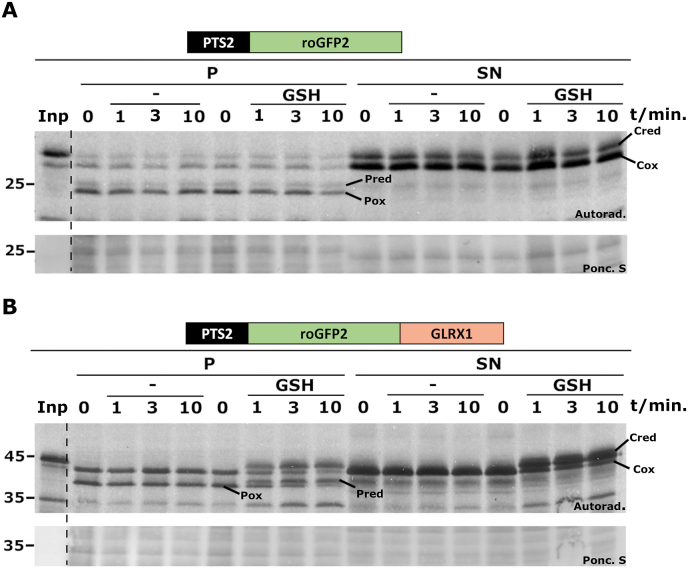
**– The peroxisomal membrane is permeable to GSH.** PNSs from import reactions containing PTS2-roGFP2 (A) or PTS2-roGFP2-GLRX1 (B) were halved, and one half was incubated with GR plus NADPH (lanes “-“) whereas the other half was incubated with GR, NADPH and GSH (lanes “GSH”). Aliquots were withdrawn at the indicated time points, and immediately pipetted into tubes containing an NEM solution (20 mM final concentration) on ice. After 15 min, samples were centrifuged, and organelle (“P”) and soluble (“SN”) fractions were analyzed by non-reducing SDS-PAGE/autoradiography. Pred and Pox - reduced and oxidized peroxisomal reporter proteins, respectively. Cred and Cox - reduced and oxidized cytosolic reporter proteins, respectively. Autoradiographs (“Autorad.”) and the corresponding ponceau S-stained membranes (“Ponc. S”) are shown; lanes Inp, radiolabeled protein used in the assays; numbers to the left indicate the molecular weight markers (in kDa).

To determine whether the peroxisomal membrane is permeable to GSH, PNSs containing oxidized PTS2-roGFP2 or PTS2-roGFP2-GLRX1 received a bolus of a mixture containing yeast glutathione reductase (GR), NADPH and GSH (5 mM final concentration). Aliquots were then removed at different time points, treated with NEM, and centrifuged to obtain the organelle and cytosolic fractions. An assay containing a mixture of GR and NADPH but no GSH, was also included in this experiment, as a control. As shown in Fig. 2A, neither the cytosolic nor the peroxisomal pool of PTS2-roGFP2 were reduced by GSH (compare lane “Inp” showing the behavior of reduced PTS2-roGFP2 with lanes “GSH”). Considering that redox equilibration of roGFP2 with the GSH/GSSG pair is mediated by glutaredoxin, both *in vivo* and *in vitro* [54], the behavior of peroxisomal PTS2-roGFP2 in this assay may suggest that peroxisomes lack a glutathione-glutaredoxin system (see also later). However, the finding that cytosolic PTS2-roGFP2 was not reduced by GSH may seem unexpected, as rat liver cytosol contains glutaredoxin. We note that the concentration of cytosolic proteins in these *in vitro* assays is ∼1/50^th^ of that *in vivo* (see Materials and Methods for details), meaning that the concentration of soluble glutaredoxin in these experiments is just 10–20 nM, a value that is probably too low to detect its activity (see below). In contrast to PTS2-roGFP2, cytosolic PTS2-roGFP2-GLRX1 was rapidly reduced by GSH, as expected (Fig. 2B, lanes “SN, GSH”). Importantly, the same result was observed for peroxisomal/processed PTS2-roGFP2-GLRX1 (Fig. 2B, Lanes “P, GSH”). Apparently, the GSH added to the PNS was able to reach the organelle matrix where it reduced peroxisomal PTS2-roGFP2-GLRX1. This finding strongly suggests that the peroxisomal membrane is permeable to GSH.

**Fig. 3:**
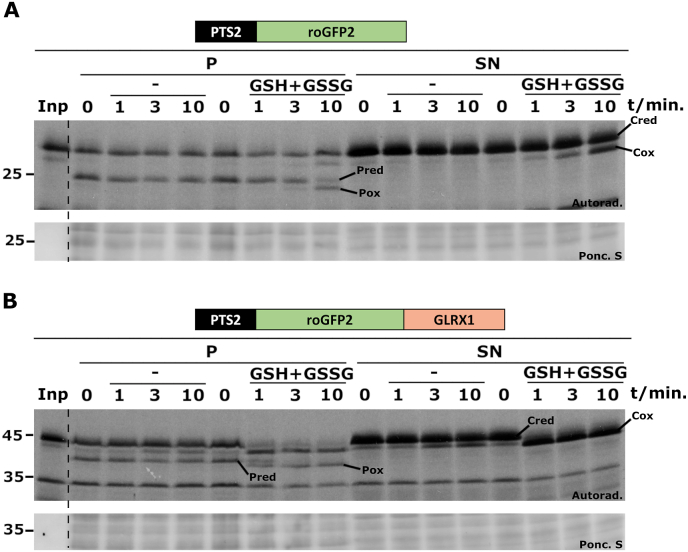
**– The peroxisomal membrane is permeable to GSSG.** PNSs from import reactions containing PTS2-roGFP2 (A) or PTS2-roGFP2-GLRX1 (B) were reduced with TCEP (400 μM, final concentration) and halved. One half was supplemented with import buffer (lanes “-“) whereas the other half received a mixture of GSH and GSSG (4.5 mM and 0.25 mM, respectively) in import buffer. Aliquots were withdrawn at the indicated time points, and immediately pipetted into tubes containing NEM. After 15 min on ice, samples were centrifuged, and organelle (“P”) and soluble (“SN”) fractions were analyzed by non-reducing SDS-PAGE/autoradiography. Pred and Pox - reduced and oxidized peroxisomal reporter proteins, respectively. Cred and Cox - reduced and oxidized cytosolic reporter proteins, respectively. Autoradiographs (“Autorad.”) and the corresponding ponceau S-stained membranes (“Ponc. S”) are shown; lanes Inp, radiolabeled protein used in the assays; numbers to the left indicate the molecular weight markers (in kDa).

The “reverse” redox experiment was also performed, that is, PNSs containing either PTS2-roGFP2 or PTS2-roGFP2-GLRX1 were reduced with TCEP and treated with a bolus of a solution comprising 4.5 mM GSH and 0.25 mM GSSG. This GSH/GSSG solution has a theoretical redox potential of – 208 mV [33], mimicking that found in the endoplasmic reticulum [55,56], and should cause complete oxidation of roGFP2 [42]. As shown in Fig. 3A, a fraction of both cytosolic and peroxisomal PTS2-roGFP2 was slowly oxidized by the GSH/GSSG solution (compare lanes “-“ with lanes “GSH + GSSG”). Considering that purified roGFP2 is also slowly oxidized by GSSG *in vitro* (*e.g.*, see Fig. 2C in Ref. [57]), oxidation of PTS2-roGFP2 in these assays probably reflects a direct reaction with GSSG. In contrast to PTS2-roGFP2, and similarly to the data shown in Fig. 2B, PTS2-roGFP2-GLRX1 was rapidly oxidized by the GSH/GSSG solution regardless of its subcellular localization (Fig. 3B, compare lanes “-“ with lanes “GSH + GSSG”). These data strongly suggest that the peroxisomal membrane is permeable also to GSSG.

**Fig. 4:**
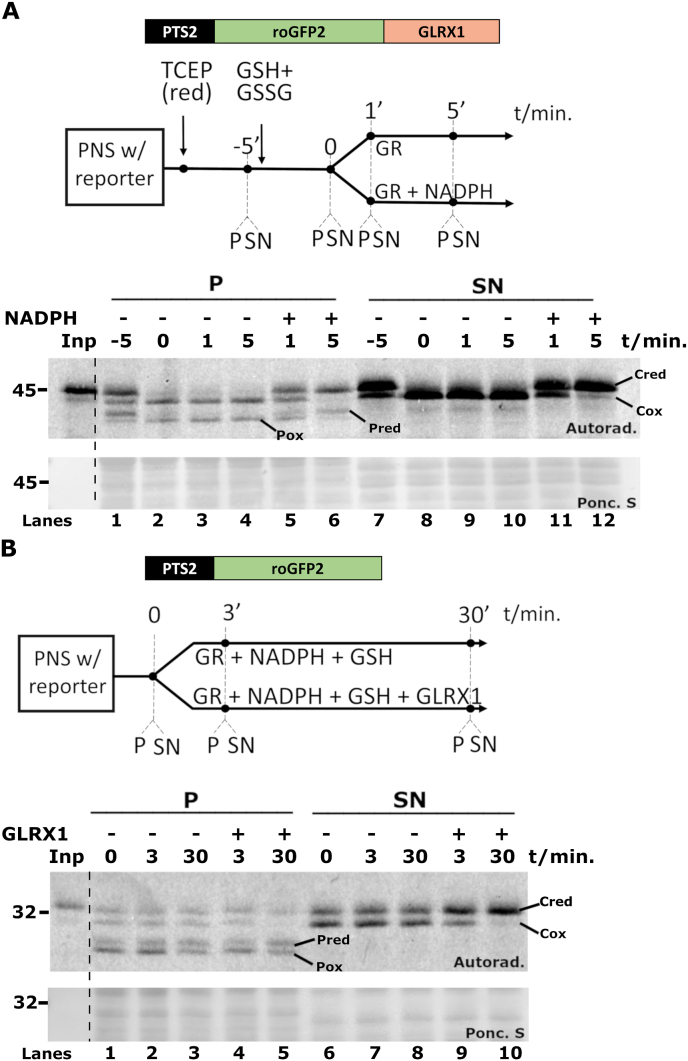
**– Oxidation/reduction of peroxisomal PTS2-roGFP2-GLRX1 is a reversible process**. **A –** A PNS containing PTS2-roGFP2-GLRX1 was reduced with TCEP (lanes 1 and 7) and supplemented with an oxidizing mixture of 4.5 mM GSH and 0.25 mM GSSG. After 5 min (lanes 2 and 8), the PNS was halved and one half was supplemented with GR alone (lanes 3, 4, 9, and 10) whereas the other half received GR plus NADPH (lanes 5, 6, 11, and 12). Aliquots were withdrawn at the indicated time points, treated with NEM, centrifuged to separate organelles (“P”) from cytosolic proteins (“SN”) and subjected to non-reducing SDS-PAGE/autoradiography. Pred and Pox - reduced and oxidized peroxisomal reporter protein, respectively. Cred and Cox - reduced and oxidized cytosolic reporter protein, respectively. **B –** The peroxisomal membrane is impermeable to GLRX1. A PNS containing PTS2-roGFP2 was halved and one half was supplemented with a mixture of GR, NADPH and GSH (lanes 2, 3, 7, and 8) whereas the other half received the same mixture plus GLRX1 (lanes 4, 5, 9, and 10). Aliquots were withdrawn at the indicated time points and processed for non-reducing SDS-PAGE/autoradiography exactly as described above. Pred, Pox, Cred and Cox - same as above. Autoradiographs (“Autorad.”) and ponceau S-stained membranes (“Ponc. S”) are shown; lanes Inp, radiolabeled protein used in the assays; numbers to the left indicate the molecular weight markers (in kDa).

Finally, we asked whether oxidation/reduction of peroxisomal PTS2-roGFP2-GLRX1 by glutathione is reversible. For this purpose, a PNS containing cytosolic and peroxisomal reporter protein was first reduced with TCEP (Fig. 4A, lanes 1 and 7) and afterwards treated with the oxidizing GSH/GSSG solution for 5 min to oxidize the reporter protein (lanes 2 and 8). Half of this sample received GR plus NADPH whereas the other half receive GR alone. As shown in Fig. 4A, both cytosolic (lanes 11 and 12) and peroxisomal PTS2-roGFP2-GLRX1 (lanes 5 and 6) were rapidly reduced in a NADPH-dependent manner indicating that a decrease in the cytosolic glutathione redox potential induced by GR and NADPH is quickly transmitted to the peroxisomal lumen.

Taken together, the data presented in Fig. 2, Fig. 3, Fig. 4A strongly suggest that the mammalian peroxisomal membrane is promptly permeable to both GSH and GSSG and that, in contrast to the situation in other organelles such as the ER and mitochondria, the peroxisomal and cytosolic pools of glutathione are redox linked.

### 2.3 The peroxisomal membrane is impermeable to cytosolic glutaredoxin 1

It is long known that during prolonged incubations under large hydrostatic pressures, such as those to which peroxisomes are subjected during purification procedures involving isopycnic ultracentrifugation, some bulky hydrophilic molecules such as iodixanol (molecular mass of 1550 Da) can permeate the peroxisomal membrane [58]. We wondered whether the peroxisomal membrane is also permeable to small proteins during prolonged incubations. Such a possibility would explain why we were unable to detect glutaredoxin activity in peroxisomes – during PNS preparation, a 30–40 min long procedure in which cytosolic proteins are diluted ∼50-fold, any glutaredoxin putatively present in the peroxisome matrix might simply diffuse out of the organelles. As no cytosolic glutaredoxin activity was detected in the redox assays above when using the PTS2-roGFP2 protein (see Fig. 2A), a similar result would be expected for the peroxisomal matrix. If this hypothesis were true, then incubating a PNS with a large amount of a glutaredoxin might result in internalization of the enzyme by peroxisomes. To test this, a PNS containing oxidized PTS2-roGFP2 was supplemented with GR, NADPH, 5 mM GSH and 5 μM of GLRX1, a small 12 kDa protein and the most abundant cytosolic glutaredoxin in mammalian cells [59]. (Note that the physiological concentration of GLRX1 in mammalian cells is around 1 μM [59] and a similar value (0.5 μM) can be estimated for rat liver from published data [60,61]. Thus, the amount of GLRX1 used in this experiment is supraphysiological). The redox state of PTS2-roGFP2 in both the soluble and organelle fractions was then assessed by non-reducing SDS-PAGE. As shown in Fig. 4B, cytosolic PTS2-roGFP2 was completely reduced in the sample containing GLRX1 after a 30 min incubation (lanes 9 and 10). In contrast, peroxisomal PTS2-roGFP2 remained oxidized under these conditions (Fig. 4B, lanes 4 and 5). We conclude that the peroxisomal membrane is impermeable to cytosolic GLRX1. Considering that thioredoxin and glutaredoxin have similar sizes, this conclusion is probably also valid for thioredoxin.

### 2.4 Kinetic simulations suggest that GSH protects peroxisomal matrix proteins from oxidation even in the absence of glutaredoxin

The data above showing that peroxisomes can import/export glutathione but lack a (detectable) glutaredoxin activity might suggest that the redox state of cysteine residues in peroxisomal proteins is not affected by the intraperoxisomal GSH/GSSG pool. Considering that H_2_O_2_ is not a particularly reactive oxidant (reviewed in Ref. [62]), and that catalase is one of the most efficient enzymes in nature [63], it might be possible that all cysteine-containing proteins that arrive at the organelle matrix maintain their reduced status for the few days they will last in a peroxisome ([64]; see also Materials and Methods). To put this idea to the test, we performed kinetic simulations.

**Fig. 5:**
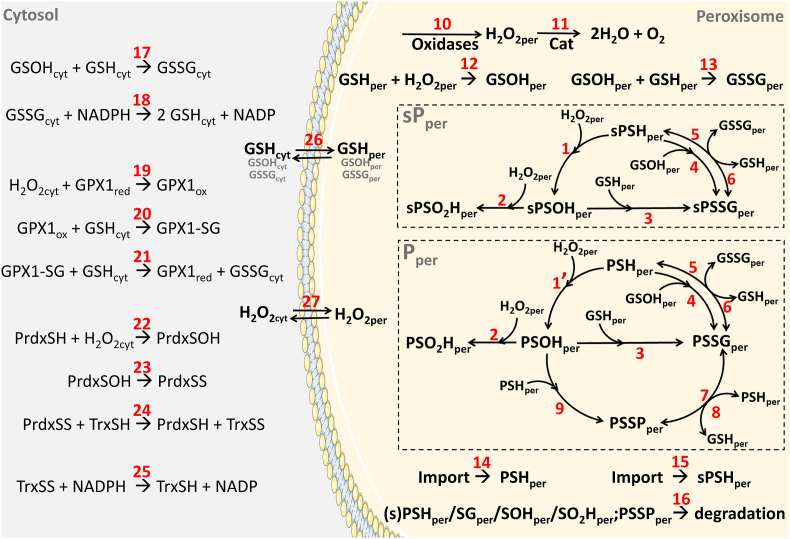
**– Schematic representation of the peroxisome-cytosol model used in kinetic simulations.** The chemical species and reactions used for kinetic simulations are indicated. Reaction rates and initial concentrations of the different species are provided in Supplementary Tables 1 and 2 The suffixes “cyt”, “per”, “red”, and “ox” stand for cytosolic, peroxisomal, reduced, and oxidized, respectively. Cat – catalase; GSH/GSSG – reduced/oxidized glutathione; GSOH –glutathione sulfenic derivative; sP_per_– hydrogen peroxide-sensitive peroxisomal protein; P_per_– total peroxisomal protein; (s)PSH_per_– reduced protein; (s)PSG_per_– glutathionylated protein; (s)PSOH_per_– protein sulfenic derivative; (s)PSO_2_H_per_– protein sulfinic derivative; PSSP_per_– protein disulfide linked; GPX1 – glutathione peroxidase 1; GPX1-SG – glutathionylated glutathione peroxidase 1; PrdxSH – reduced peroxiredoxin; PrdxSOH – peroxiredoxin sulfenic derivative; PrdxSS – peroxiredoxin disulfide linked; TrxSH – reduced thioredoxin; TrxSS – disulfide linked thioredoxin. (For interpretation of the references to colour in this figure legend, the reader is referred to the Web version of this article.)

The model built for this purpose (see Fig. 5) comprises a peroxisome immersed in a 24-fold larger cytosol, the volumetric proportion found in a rat hepatocyte [65]. The peroxisome contains catalase (Fig. 5, reaction 11) and a constant source of H_2_O_2_ (reaction 10); its membrane is permeable to both H_2_O_2_ (reaction 27) and glutathione (reaction 26). Experimental values for the rate of H_2_O_2_ generation in the rat liver peroxisome, catalase concentration, 2^nd^ order rate constant for H_2_O_2_ dismutation by the enzyme, as well as the permeability coefficient of H_2_O_2_ across the organelle membrane were obtained from the literature (see Supplementary Tables 1 and 2, and Materials and Methods). The unknown permeability coefficient of glutathione species across the peroxisomal membrane was arbitrarily set to 1/100^th^ that of H_2_O_2_ but, as shown below, decreasing this variable by several orders of magnitude does not change conclusions. A set of antioxidative enzymes comprising glutathione reductase (Fig. 5, reaction 18), glutathione peroxidase 1 (GPX1; reactions 19–21), peroxiredoxins 1/2 (reactions 22–24), thioredoxin and thioredoxin reductase (reactions 24 and 25) was placed in the cytosol to consume the H_2_O_2_ and GSSG that exit the peroxisome.

Two different types of cysteine-containing reporter proteins were placed inside the organelle. One, hereafter referred to as sPper, comprises ∼2% of the total peroxisomal matrix protein (*i.e.*, [sPper] = 100 μM), and possesses a reactive cysteine that reacts with H_2_O_2_ (Fig. 5, reaction 1) with a rate constant of 100 M^−1^ s^−1^, a value similar to those described for catalytic cysteine residues of some phosphatases, papain and glyceraldehyde-3-phosphate dehydrogenase [[66], [67], [68], [69]]. The sulfenylated protein (sPSOHper) can be further oxidized by H_2_O_2_ to the sulfinyl derivative (sPSO_2_Hper; reaction 2) or glutathionylated (sPSSGper; reaction 3). Reduced sPper can also be glutathionylated by reaction with sulfenylated glutathione (reaction 4) or undergo reversible thiol-disulfide exchange with GSSG (reactions 5 and 6). It is assumed that the oxidation-prone cysteine residue of sPper resides in a protein cavity and thus is not able to form a disulfide bridge with other proteins. Although no experimentally determined rate constants for oxidation of a peroxisomal matrix protein by H_2_O_2_ are presently available, sPper might be ACAA1a or SCPx, two peroxisomal thiolases that contain oxidation-prone cysteine residues [70,71]. The other reporter protein, referred to as Pper, corresponds to the total peroxisomal matrix protein. Its concentration in the organelle matrix is thus larger (4.6 mM; see Materials and Methods). It is assumed that Pper contains a single exposed cysteine residue, the thiol group of which can undergo the oxidation reactions described above for sPper (see Fig. 5). However, unlike sPper, oxidized Pper (*i.e.*, PSOHper and PSSGper) can also form an intermolecular disulfide bridge with PSHper (Fig. 5, reactions 8 and 9). (Note that this is a likely scenario because several peroxisomal matrix proteins yield high-molecular weight products when treated with diamide, indicating that their cysteine residues can engage in intermolecular disulfide bonds; see Fig. S1). The single cysteine residue in PSHper reacts slowly with H_2_O_2_ (Fig. 5, reaction 1’) as most non-catalytical cysteines do, with a second order rate constant of 2.7 M^−1^ s^−1^, a value we adopted from the reaction of Human Serum Albumin (HSA) with H_2_O_2_ [72]. Rate constants for thiol-disulfide exchange (1 M^−1^ s^−1^; Fig. 5, reactions 5–8), reaction between cysteine-sulfenic and cysteine thiol (10 M^−1^ s^−1^; Fig. 5, reactions 3, 4, 9, 13 and 17) and sulfinylation of cysteine-sulfenic acid (100 M^−1^ s^−1^; reaction 2) were used in the model (see Materials and Methods for details). Finally, because simulations were performed using a time window of 48 h, rates of peroxisomal protein degradation (average half-life (t_1/2_) = 39 h; Fig. 5, reaction 16) and replenishment (import; reactions 14 and 15) were also considered.

Supplementary Fig. S2 shows a sensitivity analysis of the model, *i.e*., how sensitive are the steady-state concentrations of the different species in the system to alterations of the kinetic parameters. As expected, the two most important parameters are the flux of H_2_O_2_ production in the peroxisome and the reaction of catalase, which have strong positive and negative effects, respectively, on virtually all oxidized species. A detailed analysis of how catalase influences the steady-state concentrations of several peroxisomal species is presented in Supplementary Fig. S3. It is clear from this analysis that maintaining low concentrations of H_2_O_2_ and GSSG in the organelle requires very large concentrations of catalase – enzyme concentrations below 10 μM provide almost no protection from these oxidative species. The sensitivity analyses shown in Fig. S2 also revealed a less intuitive result: the permeability of the peroxisomal membrane to H_2_O_2_ has only a minor effect on the peroxisomal concentration of this species. Indeed, the steady-state concentration of peroxisomal H_2_O_2_ in the reference conditions (76 nM; see Supplementary Table 3) increases to just 81 nM when the permeability constant of H_2_O_2_ is set to 0. Apparently, the catalytic efficiency and large concentration of catalase in peroxisomes would be sufficient to neutralize all the H_2_O_2_ that is generated in the organelles. Another interesting prediction regards the importance of peroxisomal catalase on the amount of NADPH consumed by the cytosolic NADPH-dependent reductive systems that neutralize the H_2_O_2_ that escapes from the organelle. The presence of catalase in the peroxisome decreases to 6% the amount of NADPH that would be otherwise consumed by the cytosolic enzymes (see Supplementary Table 3). Clearly, the kinetic model described here can provide mechanistic information on several aspects of peroxisome redox homeostasis.

**Fig. 6:**
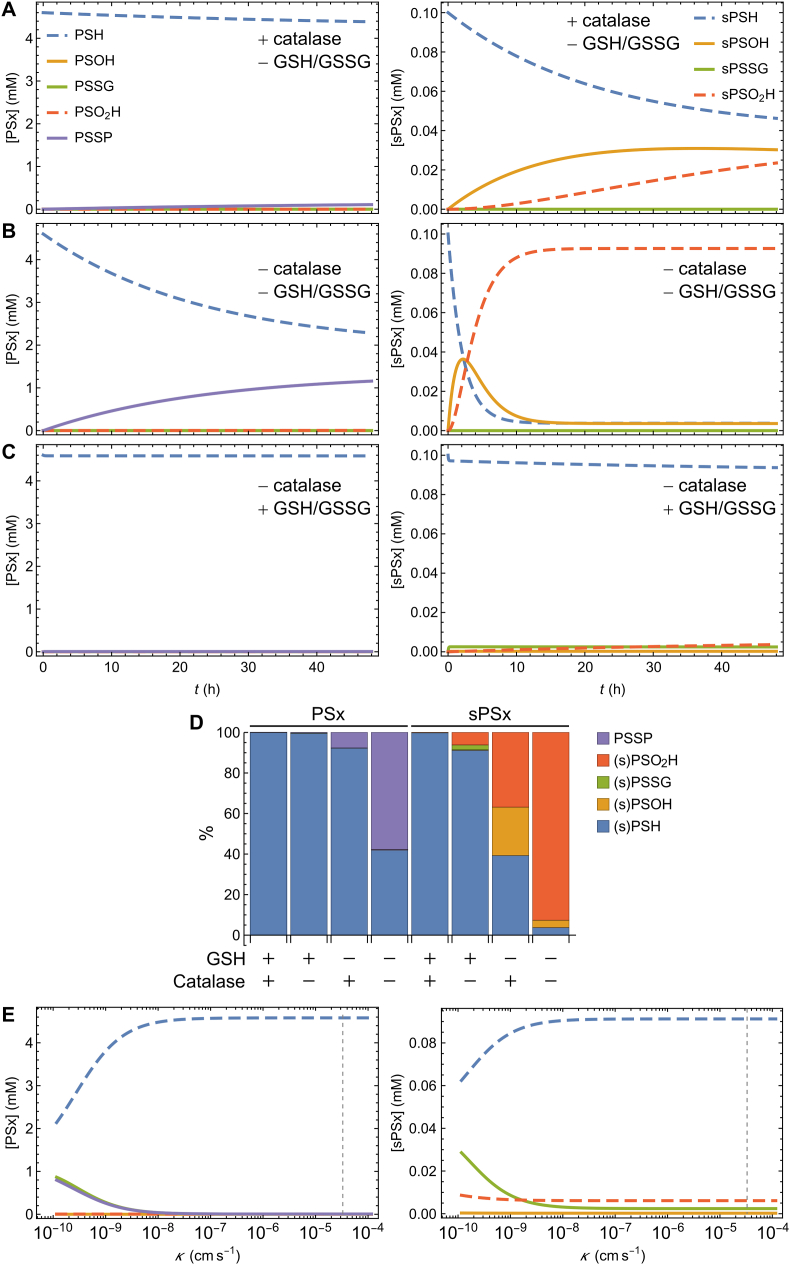
**– Kinetic simulations of protein oxidation in peroxisomes.** Simulations were performed with a peroxisome containing catalase but not glutathione (A), or lacking catalase and glutathione (B), or lacking catalase but with a membrane permeable to GSH/GSSG (C). D – levels of all protein species expressed in monomeric units at steady-state. E − redox state of the two reporter proteins as a function of the permeability coefficient (K) of glutathione species. The dashed vertical line indicates the permeability coefficient used in the simulations. PSH, PSOH, PSO_2_H, PSSG, PSSP represent pools of total peroxisomal protein in reduced, sulfenic, sulfinic, glutathionylated and dimeric state, respectively; sPSH, sPSOH, sPSO_2_H, sPSSG represent pools of hydrogen peroxide-sensitive peroxisomal protein in reduced, sulfenic, sulfinic, and glutathionylated state, respectively; PSx and sPSx indicate total peroxisomal protein and hydrogen-peroxide sensitive peroxisomal protein in all possible redox states, respectively.

To assess the potential importance of glutathione to peroxisomal protein redox homeostasis, we started by analyzing a system containing a peroxisome that cannot exchange glutathione species with the cytosol (*i.e.*, the membrane permeability constants for all glutathione species and their intraperoxisomal concentrations are 0) and where all the redox pools are initially fully reduced. Under these conditions, 95% of Pper reaches the end of the simulation in the reduced state (Fig. 6A, left panel), in agreement with the hypothesis above. In contrast, more than 50% of sPper is oxidized by H_2_O_2_ (Fig. 6A, right panel), suggesting that either such a H_2_O_2_-sensitive protein does not exist in peroxisomes or, if it does, that an intraperoxisomal antioxidant system relying solely on catalase and diffusion of peroxisomal H_2_O_2_ to the cytosol is not sufficient to maintain H_2_O_2_-sensitive proteins in a reduced state. Data supporting the latter possibility were obtained when the peroxisomal catalase activity was set to 0, a condition that mimics a peroxisome from a catalase-knockout mouse. Under these conditions, 50% of Pper (Fig. 6B, left panel) reaches the end of the simulation as disulfide-linked dimers, an unlikely situation when we consider that the catalase-knockout mouse has normal peroxisomal functions ([27]; see also [73]) and that peroxisomes from this mouse have an unaltered matrix thiol-disulfide redox potential [28].

Remarkably, when glutathione species are allowed to permeate the membrane of a peroxisome that lacks catalase, 99.7% of Pper and 94% of sPper remain in the reduced state (Fig. 6C, left and right panels, respectively). Over time, a steady state is eventually reached. Fig. 6D shows the percentages of the various forms of the Pper and sPper proteins at steady state in each of the conditions discussed above. Of note, catalase has a modest effect on Pper and sPper oxidation when GSH is available and able to permeate the peroxisomal membrane.

Importantly, both proteins remain strongly reduced in a catalase-less peroxisome even when the membrane permeability coefficients of the glutathione species are decreased by 4 orders of magnitude, *i.e.,* to ∼3.3 × 10^−9^ cm s^−1^ (Fig. 6E; for comparison, note that the permeability coefficient of glucose in a pure phospholipid membrane (*i.e.*, with no transporters) is ≤ 10^−10^ cm s^−1^, whereas that in a human erythrocyte membrane is ∼10^−4^ cm s^−1^ [74]). Under these conditions, 93% of Pper and 91% of sPper remain fully reduced (Fig. 6E; PSH e sPSH, respectively). We conclude that even in the absence of a glutaredoxin, glutathione is an important component of the peroxisomal matrix antioxidant defense.

## 3 Conclusions

The presence of GSTk1 in mammalian peroxisomes was until now the only evidence supporting the hypothesis that the organelle membrane is permeable to GSH (reviewed in Ref. [75]). However, as pointed out in the Introduction, the type of reaction catalyzed *in vivo* by GSTk1 is still unknown, and thus the possibility that the glutathione used by GSTk1 reaches the peroxisomal matrix already bound to the enzyme cannot be formally excluded. But even if we assumed that GSTk1 uses GSH as a substrate, and thus that the peroxisomal membrane is permeable to GSH, we would still not know whether the same is valid for GSSG and whether the peroxisomal and cytosolic pools of glutathione are in redox equilibrium. Actually, data on peroxisomes from other organisms might lead us to conclude otherwise. Indeed, in contrast to mammalian peroxisomes, both yeast and plant peroxisomes have been shown to contain a fraction of cytosolic glutathione reductase [76,77], a finding that may suggest that any GSSG formed in the peroxisomal lumen of those organisms cannot be promptly exported to the cytosol.

In this work we provide data indicating that the mammalian peroxisomal membrane is permeable to both GSH and GSSG and that the intraperoxisomal and cytosolic pools of glutathione are redox linked. Thus, one of the two conditions required to have an operating glutathione-glutaredoxin-based system inside peroxisomes is fulfilled. Unexpectedly, however, we were unable to detect glutaredoxin activity in the organelles. We still considered the possibility that the peroxisomal membrane might be weakly permeable to small proteins such as GLRX1 upon prolonged incubations. However, as shown in Fig. 4B, this turned out not to be the case.

Although these results are in line with most proteomic studies suggesting that mammalian peroxisomes do not contain a glutaredoxin, it may be premature at present to completely exclude such a possibility. Indeed, although mammalian glutaredoxins lack canonical peroxisomal targeting signals, small amounts of one of these proteins may still be imported into peroxisomes, for instance by a piggy-back import mechanism, as previously described for superoxide dismutase 1 [11]. Considering the size of a typical liver peroxisome (diameter of 0.7 μm) it would suffice to import ∼10 molecules of glutaredoxin to reach a concentration of 0.1 μM in the organelle lumen. Such a concentration is 50-fold lower than that used in the experiment shown in Fig. 4B, and thus the corresponding glutaredoxin activity would have been easily missed.

Regardless of this uncertainty, the kinetic simulations described here suggest that the intraperoxisomal pool of glutathione plays an important role in peroxisomal redox homeostasis even in the absence of a glutaredoxin. Indeed, we found that the presence of glutathione in the peroxisomal matrix is more important to keep protein cysteine residues in a reduced state than catalase itself (Fig. 6, compare panels A and C). It is evident from these simulations that this is possible only because 1) GSH is an abundant species, 2) the steady-state concentration of H_2_O_2_ inside peroxisomes, as estimated from the kinetic simulations, is relatively low (∼76 nM), even in an organelle that lacks catalase (∼1.3 μM) due to the large permeability of the peroxisomal membrane to H_2_O_2_, and, more importantly, 3) although H_2_O_2_ is a strong oxidant, it reacts very slowly with most protein cysteine residues. Naturally, more reactive ROS will increase the oxidation rates of peroxisomal proteins, a possibility that we did not explore here because there is presently no information on the concentrations of other ROS inside peroxisomes. Nevertheless, it is worth noting that for ROS that oxidize cysteine residues to the sulfenyl derivatives (*e.g.*, organic hydroperoxides, peroxynitrite [3]) the glutaredoxin-independent action of GSH still protects ∼80% of intraperoxisomal proteins from oxidation as long as the ROS concentration times the second-order rate constant of its reaction with protein cysteine residues is ≤ ∼ 5 × 10^−4^.s^−1^. For instance, peroxynitrite reacts with the thiol group of HSA with a second-order rate constant that is 3 orders of magnitude larger than that of the reaction between the protein and H_2_O_2_ (3.8 × 10^3^ M^−1^ s^−1^ and 2.7 M^−1^ s^−1^, respectively [72,78]). Assuming a similar rate constant for the reaction between PSHper and peroxynitrite, the intraperoxisomal pool of glutathione would still protect ∼87% of PSHper from oxidation even if the concentration of peroxynitrite in the organelle was equal to that of H_2_O_2_, *i.e.*, 76 nM.

In summary, the data described here strongly suggest that the mammalian peroxisomal membrane is permeable to both GSH and GSSG and that the intraperoxisomal and cytosolic pools of glutathione are in redox equilibrium, two findings that are important for our understanding of the redox homeostasis mechanisms that operate in the mammalian peroxisome. In addition, we developed a kinetic model that provides mechanistic insight on how peroxisomes maintain their cysteine-containing proteins in the reduced state.

## 4 Materials and Methods

### 4.1 Plasmids

Plasmids pQE30-His-PEX14 (1–80) [79] encoding an N-terminally His-tagged protein that comprises residues 1–80 of human PEX14 (referred to as NDPEX14), pET28a-His-PEX5 (1–324) that encodes an N-terminally His-tagged protein comprising residues 1–324 of human PEX5 (referred to as PEX5 (1–324) [80]) and pET28a-His-PEX7 which encodes a N-terminally tagged version of human PEX7 [47] have been described before.

The following plasmids were generated:

pET23a-PTS2-roGFP2-GLRX1: This plasmid encodes a chimeric protein comprising a N-terminal peptide that contains the PTS2 pre-sequence of human phytanoyl-CoA hydroxylase (MVDNNNNEQLRAAARLQIVLGHLGRPSAGAVVAHPTSGTSSGFPEAASSFRTHQVSGS) followed by roGFP2, a 34 amino acid-long flexible linker (TSGGS (GGGGS)_5_GGEF), and finally, human GLRX1 [43]. The corresponding cDNA was synthesized by GenScript and cloned into pET23a using the restriction sites NdeI/HindIII.

pET23a-PTS2-roGFP2: This plasmid encodes a chimeric protein comprising the N-terminal PTS2 peptide described above and roGFP2. It was obtained by site-directed mutagenesis using the plasmid pET23a-PTS2-roGFP2-GLRX1 and the primers FW_T297Stop (GGCATGGACGAGCTGTACAAGTGACTAGTGGTGGTTCAGGTGG) and RV_T297Stop (CCACCTGAACCACCACTAGTCACTTGTACAGCTCGTCCATGCC), which introduce a stop codon at the end of the roGFP2 sequence.

pET28a-His-TEV-GLRX1: This plasmid encodes a histidine tag, a TEV cleavage site and human GLRX1. This plasmid was generated by GenScript by cloning the human cDNA encoding human GLRX1 (NCBI accession number NM_002064.3) into the pET-28a-TEV plasmid using the NdeI/EcoRI restriction sites.

### 4.2 Expression and purification of recombinant proteins

Recombinant PEX5 (1–324) [80] and His-NDPEX14 [79], were obtained as described before. Expression of His-tagged GLRX1 was performed in the BL21 strain of *Escherichia coli* as described [79]. Pelleted cells were cooled on ice, resuspended in a buffer containing 20 mM phosphate buffer (pH 8.0), 150 mM NaCl, 0.1% (w/v) Triton X-100, 0.1 mg/mL phenylmethylsulfonyl fluoride (PMSF) and 1:500 (v/v) protease inhibitor cocktail (Sigma-Aldrich, cat. No. P8340) supplemented with 200 μg/mL lysozyme and incubated on ice for 30 min. Cells were disrupted by sonication (Branson sonifier 250, equipped with a macro tip; 10 × 12 s, 40% duty cycle, 10% potency) and clarified by centrifugation at 16 000×*g*, 4 °C. The supernatant was incubated with Ni Sepharose 6 Fast Flow affinity chromatography beads (GE Healthcare) for 2 h, at 4 °C with gentle agitation. The protein was purified according to the manufacturer's instructions with the exception that the elution step was performed with 400 mM imidazole in 20 mM phosphate buffer (pH 8.0), 150 mM NaCl. The eluted protein was concentrated and the buffer exchanged to 50 mM Tris-HCl (pH 7.5), 150 mM NaCl, 1 mM EDTA-NaOH (pH 8.0) using a Vivaspin PES 2 concentrator MWCO 10 kDa (GE Healthcare). The N-terminal His-tag was removed by digestion with His-tagged TEV protease at an enzyme:protein ratio of 1:10, overnight at 4 °C. The digest was then incubated with HIS-Select Nickel Affinity Gel beads (Sigma-Aldrich) for 2 h at 4 °C, and the cleaved protein was recovered from the non-bound fraction. Note that the TEV protease leaves two extra amino acid residues (Gly and His) preceding the initial methionine of GLRX1. The protein was then concentrated, and the buffer exchanged to 50 mM Tris-HCl (pH 8.0), 150 mM NaCl by repeated cycles of centrifugation and dilution using a Vivaspin PES 500 concentrator MWCO 10 kDa (GE Healthcare). Recombinant GLRX1 was stored at −80 °C.

### 4.3 *In vitro* synthesis of ^35^S-labeled proteins

Plasmids encoding PTS2-roGFP2 or PTS2-roGFP2-GLRX1 proteins were used as templates in in *vitro* transcription/translation reactions using the TNT® T7 Quick Coupled Transcription/Translation System (Promega) in the presence of EasyTag™ L-[^35^S]methionine (specific activity >1000 Ci/mmol, PerkinElmer) for 90 min at 30 °C, according to the manufacturer instructions. The rabbit reticulocyte lysates (RRL) containing the *in vitro* synthesized proteins were aliquoted and stored at −80 °C. A plasmid encoding histidine-tagged human PEX7 (His-PEX7) was also used as template in *in vitro* transcription/translation reactions using the TNT® T7 Quick Coupled Transcription/Translation System (Promega). In this case, the radioactive methionine was replaced by 20 μM unlabeled methionine and the reaction was incubated for 4 h at 30 °C, conditions that increase the amount of soluble (folded) PEX7. Aliquots were stored at −80 °C.

### 4.4 Cell-free *in vitro* import assays

Male Wistar Han rats, with 6–8 weeks of age, were euthanized following the guidelines/protocols approved by the IBMC Animal Ethics Committee (CEA – Comissão de Ética Animal). All experimental protocols were approved by the Portuguese General Veterinarian Board (DGAV–Direção Geral de Alimentação e Veterinária). Rat liver post-nuclear supernatant (PNS) for *in vitro* assays was prepared as described before [41]. The trimeric protein complex comprising the radiolabeled PTS2 protein, PEX7 and PEX5 (1–324) was preassembled by mixing 1 μL of the RRL containing the radiolabeled PTS2 protein with 1 μL of a 200 ng/μL solution of recombinant PEX5 (1–324) in 50 mM Tris-HCl (pH 8.0), 150 mM NaCl, and 2 μL of the His-PEX7-containing RRL and incubating the mixture at 23 °C for 15 min [47]. *In vitro* import assays were performed exactly as described before [41] with the exception that GSH was omitted from the import buffer (glutathione is used only later in the redox assays; see below). Under these conditions, the peroxisomal and cytosolic pools of the two reporter proteins used in this work reach the end of the import reaction mostly oxidized. A standard *in vitro* import reaction (100 μL final volume) contains 500 μg of PNS (protein) in import buffer (50 mM MOPS-KOH (pH 7.4, at 37 °C), 50 mM KCl, 250 mM sucrose, 5 mM MgCl_2_), 3 mM ATP, 10 μM bovine ubiquitin and 1 μL of the RRL mixture containing the preassembled trimeric complex. Reactions were incubated at 37 °C for 20 min and import was blocked by adding 10 μM (final concentration) of NDPEX14 and incubating at 37 °C for an additional 2 min. These PNSs were then immediately used in the redox assays described below. In some experiments, import reactions were subjected to a protease-protection assay. Specifically, at the end of the 37 °C incubation, samples were put on ice, halved, and proteinase K (400 μg/mL final concentration) was added to one of the samples. After 20 min, PMSF (500 μg/mL; 5 min on ice) was added to all samples to inhibit the protease where present. Samples were then diluted 1:10 (v/v) with ice-cold SEMK (0.25 M sucrose, 20 mM MOPS-KOH (pH 7.4), 1 mM EDTA-NaOH (pH 8.0), 50 mM KCl), and centrifuged at 16 000×*g*, 20 min, at 4 °C to separate organelles from cytosolic/soluble proteins. Both fractions were precipitated with 10% (w/v) trichloroacetic acid, washed with acetone and solubilized with Laemmli sample for 15 min at 65 °C and then for 5 min at 90 °C. Total organelle pellets (from 500 μg of PNS protein) and 1/10^th^ or 1/20^th^ of the cytosolic fractions were loaded onto the SDS-gels.

### 4.5 Redox assays

PNSs subjected to *in vitro* import assays were used in the redox experiments. For the reduction assays, the PNSs were used directly since most of the reporter proteins are already oxidized. Specifically, import reactions were mixed with an equal volume of a pre-warmed solution (2 min at 37 °C) comprising 2 mM NADPH and 1.5 U of yeast glutathione reductase (GR; Merck, cat. G3664) containing or not 10 mM GSH, and incubated at 37 °C. Aliquots containing 500 μg PNS protein were withdrawn at different time points into tubes placed on ice and already containing 100 mM NEM (20 mM NEM, final concentration). After 10 min, samples were processed for SDS-PAGE analyses, as described above.

For the oxidation assays, the PNSs were first reduced with 400 μM TCEP (final concentration; added from a 50 mM stock solution in water, pH ∼7 with NaOH) for 5 min at 37 °C. The reactions were then diluted with an equal volume of either import buffer or a solution of 9 mM GSH and 0.5 mM GSSG in import buffer and incubated at 37 °C. Samples were withdrawn at different time points into ice-cold tubes containing 100 mM NEM (20 mM NEM, final concentration) and processed for SDS-PAGE analyses, as above.

For the reduction assays in the presence of GLRX1, PNSs containing the radiolabeled PTS2-roGFP2 protein were incubated at 37 °C with 5 mM GSH, 1 mM NADPH, and 0.8 U GR (final concentrations) in the presence or absence of 5 μM recombinant GLRX1. Aliquots containing 500 μg of PNS protein were withdrawn at different time points into tubes containing NEM and processed for SDS-PAGE analyses, as above.

### 4.6 Kinetic simulations

The model comprises a 1 L peroxisome in a 24 L cytosol, the volumetric proportion found in a rat hepatocyte [65]. The large peroxisome has the surface/volume ratio of an average peroxisome in rat liver (radius of 0,35 μm [81]; surface/volume ratio (3/r) = 8.6 × 10^4^ cm^−1^) and thus its surface is 8.6 × 10^7^ cm^2^.

The rate of H_2_O_2_ production in liver peroxisomes (measured in anesthetized rats with endogenous substrates) is 380 nmol/min per g of liver [82]. Considering that the volumic mass of liver is 1.07 g/mL [83] and that peroxisomes correspond to 1.89% of total liver volume [65] the rate of H_2_O_2_ production in peroxisomes is thus 359 μM s^−1^.

The protein content of rat liver is 206 mg/g wet weight [84] or 220.4 mg/mL of liver. Peroxisomes comprise 1.92% of the total liver protein [84] and 1.89% of the organ volume. Thus, the protein concentration in peroxisomes is 224 g/L. Considering that the average peroxisomal protein has a molecular mass of 49 kDa [85] the total protein concentration in peroxisomes is 4.6 mM. Catalase comprises 15% of the total peroxisomal protein [7] and its molecular mass (monomer) is 60 kDa. Thus, the concentration of catalase (monomer) in peroxisomes is 560 μM.

The permeability coefficient of H_2_O_2_ in the peroxisomal membrane is 3.3 × 10^−3^ cm s^−1^ [1]. The permeability constant of the 1 L peroxisome to H_2_O_2_ is thus 8.6 × 10^7^ cm^2^ x 3.3 × 10^−3^ cm s^−1^ = 284 L/s.

The second order rate constant determined for bovine liver catalase, 7.9 × 10^6^ M^−1^ s^−1^ [86] was used in the simulations. Second order rate constants for the reaction between H_2_O_2_ and noncatalytic cysteine residues was 2.7 M^−1^ s^−1^ for PSHper, based on the value for HSA [72] and 0.87 M^−1^ s^−1^ for GSH [29]. For active-site cysteine residues, second order rate constants for their oxidation by H_2_O_2_ are quite variable ranging from 10 to 500 M^−1^ s^−1^ [3,62,66,87]. We used a value of 100 M^−1^ s^−1^ for the oxidation of sPSHper by H_2_O_2_. Second order rate constants for the oxidation of cysteine sulfenic to sulfinic acid by H_2_O_2_ in several proteins is 60–110 M^−1^ s^−1^ at 20 °C, pH 7.0–8.0 [66,88]. We used a value of 100 M^−1^ s^−1^ for this reaction. Nonenzymatic thiol-disulfide exchange is a slow reaction with rate constants in the range 0.1–10 M^−1^ s^−1^ [29]. A value of 1 M^−1^ s^−1^ was used in the simulations. Using the lower value (*i.e.*, 0.1 M^−1^ s^−1^) does not change the conclusions of the simulations although the amount of sPSHper remaining after 48 h in a peroxisome lacking catalase is ∼75% (instead of 94%); the decrease in sPSHper is due mainly to an increase in sPSSGper, *i.e.*, GSH still protects sPSHper from overoxidation. Cysteine sulfenic acid reacts extremely fast with cysteine thiol group - the second order rate constant for this reaction is > 10^5^ M^−1^ s^−1^ [29]. To the best of our knowledge there is only one rate constant for the reaction between a protein-sulfenic acid and GSH available in the literature. The protein is HSA and the rate constant is 2.9 M^−1^ s^−1^ at pH 7.4, 25 °C [72]. Assuming a Q_10_ = 2, the rate constant at 37 °C is 6.7 M^−1^ s^−1^. However, as noted in Ref. [29], the cysteine residue in HSA is in a hydrophobic cavity, which explains the stability of its sulfenic acid. We used a rate constant of 10 M^−1^ s^−1^ for the reaction between cysteine-sulfenic acid and the cysteine-thiol group, a value that is conservative.

The concentration of GPX1 in rat liver cytosol is 7.6 μM, as calculated from the data in Refs. [61,89]. The rate constants for the reduction of H_2_O_2_ by GPX1 (a 3-step process) are 2.7 × 10^7^ M^−1^ s^−1^ for the GPX1 oxidation, 4 × 10^4^ M^−1^ s^−1^ for the glutathionylation and 1 × 10^7^ M^−1^ s^−1^ for the reduction of GPX1 [90]. Rat liver contains 10 U/g of glutathione reductase [91], or 10.7 U/mL (V_max_ = 1.78 × 10^−4^ M s^−1^). The rate law for glutathione reductase (ping pong mechanism) is provided in Ref. [92] (K_m(NADPH)_ = 7.9 × 10^−6^ M; K_m(GSSG)_ = 5.67 × 10^−5^ M). The NADPH concentration in rat liver cytosol is 360 μM [93].

To simplify the modeling of the clearance of H_2_O_2_ by the cytosolic 2-Cys peroxiredoxins PRDX1 and PRDX2, we aggregated these two peroxiredoxins in a single species, as previously described in Ref. [94]. Thus, the rate constant for each process is a weighted average of the rate constants determined for human PRDX1 and PRDX2, the weights reflecting these proteins’ relative abundances in human hepatocytes as estimated in Ref. [94]. The catalytic cycle of these enzymes occurs in three steps. In the first, H_2_O_2_ reacts with the so-called peroxidatic Cys forming a sulfenic acid. Rate constants determined for this step are in the range of (1.3–16) × 10^7^ M^−1^ s^−1^, similar for both proteins [[95], [96], [97], [98]]. We adopted the value 1.0 × 10^7^ M^−1^ s^−1^. The sulfenic acid then condenses with a proximal Cys forming a disulfide. Rate constants for this step are in the 9–12.9 s^−1^ range for PRDX1 and 0.2–0.64 s^−1^ for PRDX2 [[97], [98], [99]]. We adopted the value 8.7 s^−1^. The disulfide is reduced by Trx1, with rate constants 2.8 × 10^6^ M^−1^ s^−1^ and 5.19 × 10^5^ M^−1^ s^−1^ for PRDX1 and PRDX2, respectively (Ana Denicola, personal communication). We adopted the value 2.3 × 10^6^ M^−1^ s^−1^. Trx1 that is oxidized in this process is in turn reduced by NADPH in a reaction catalyzed by TrxR. We adopted the *V*_max_ = 50 μM s^−1^ estimated for human hepatocytes in Ref. [94] and *K*_M_(Trx1) = 1.8 μM [100], and we assumed that the enzyme was saturated with NADPH, considering its low *K*_M_(NADPH) = 6 μM ([101]; see Ref. [94] for further details).

The half-life (t_1/2_) of rat liver catalase, as assessed by decay of its heme group, is 44 h [102]. Similar values have been found for several peroxisomal proteins in mouse liver (*e.g.*, Abcd3 (t_1/2_ = 31.4 h), Hsd17b4 (t_1/2_ = 51.9 h), Scp2 (t_1/2_ = 28.9 h), Acaa1a (t_1/2_ = 22.4 h), Acox1 (t_1/2_ = 41.9 h), Acox2 (t_1/2_ = 56.0 h), Decr2 (t_1/2_ = 44.9 h), Ehhadh (t_1/2_ = 41.9 h); Cat (t_1/2_ = 25.0 h); and UOX (t_1/2_ = 45.3 h); [103]). The average t_1/2_ of these 11 proteins is 39 h, the value used in the model. The specific rate of degradation (K_degr_ = ln2/t_1/2_) is thus 4.937 × 10^−6^ s^−1^. For simplicity, we assume that protein degradation is a random first order event affecting all populations of a given protein (*e.g.*, PSHper, PSOHper, and PSO_2_Hper), and that new protein (*i.e.*, PSHper and sPSHper) enters the peroxisome (import) at the same specific rate (*i.e*., V_imp_ = K_degr_ x (total protein concentration of Pper or sPper)), to keep the total protein concentration constant. When protein degradation and import is removed from the simulations, the percentage of oxidized Pper in a peroxisome lacking both catalase and glutathione increases by 37%.

Equations and rate constants used in the simulations are provided in Table S1. Initial concentrations of the different species are provided in Table S2. Differential equations are shown in Fig. S4. We ran all the simulations in Copasi v. 4.39 (Build 272) [108] using LSODA with relative tolerance 10^−6^, absolute tolerance 10^−12^, 100 000 max internal steps and 0 max internal step size. For computing steady states, we used the combined integration and Newton methods, *i.e.*, the equations were first integrated (with the above settings) until a steady state was approached, and the concentration values attained were then used as initial guesses for Newton's method to polish the steady state by numerically solving the (algebraic) steady-state equations. The relevant settings were: resolution 8 × 10^−7^, derivation factor 0.001, iteration limit 100, Maximum duration of forward integration 10^9^, and target criterion rate.

### 4.7 Miscellaneous

The dilution factor of rat liver cytosolic proteins in the redox assays (∼1/50) was estimated considering that ∼2/3 of the PNS protein represents soluble/cytosolic protein (*i.e.*, 333 μg/100 μL or 0.333% (w/v)) and that the *in vivo* concentration of cytosolic protein in rat liver cells is ∼17.5% (w/v) [61]. Antibodies to ACAA1a [104] and ACOX1 [105] were kindly provided by Prof. Marc Fransen, KU-Leuven, Belgium. Antibodies directed to catalase (Research Diagnostics, Inc., cat. No. RDI-CATALASEabr) and SCPx (Proteintech, cat. no. 14397-1-AP) were purchased. Primary antibodies were detected in western-blots with goat alkaline phosphatase-conjugated anti-rabbit antibodies (Sigma-Aldrich, cat. no. A9919).

## Authorship

MJF – Conception, design, data acquisition, analysis and interpretation, drafting and revising of the article.

JEA, TF – Conception, design, data analysis and interpretation, drafting, and revising of the article.

TAR, AGP – Data acquisition, analysis and interpretation, and revising of the article.

AS, LG – Data analysis and interpretation, and revising of the article.

All authors have contributed to the manuscript and approved the submitted version.

## Declaration of competing interest

The authors declare that they have no known competing financial interests or personal relationships that could have appeared to influence the work reported in this paper.

## Data Availability

No data was used for the research described in the article.
