# Supplementary material for: The mammalian peroxisomal membrane is permeable to both GSH and GSSG – Implications for intraperoxisomal redox homeostasis
Source: Redox Biol. 2023 May 25;63:102764. doi: 10.1016/j.redox.2023.102764 (PMC10245115; doi:10.1016/j.redox.2023.102764)
Supplement: Multimedia component 1 [file mmc1.pdf]

Supplementary Information for:

The mammalian peroxisomal membrane is permeable to both GSH and GSSG – implications for intraperoxisomal redox homeostasis.

Maria J. Ferreira[1,2,3], Tony A. Rodrigues[1,2,3], Ana G. Pedrosa[1,2,3], Luís Gales[1,2,3], Armindo Salvador[4,5,6], Tânia Francisco[1,2,3,#], and Jorge E. Azevedo[1,2,3, #]

[1]Instituto de Investigação e Inovação em Saúde (I3S), Universidade do Porto, Rua Alfredo Allen, 208, 4200-135 Porto, Portugal

[2]Instituto de Biologia Molecular e Celular (IBMC), Universidade do Porto, Rua Alfredo Allen, 208, 4200-135 Porto, Portugal

[3]Instituto de Ciências Biomédicas de Abel Salazar (ICBAS), Universidade do Porto, Rua de Jorge Viterbo Ferreira, 228, 4050-313 Porto, Portugal

[4]Coimbra Chemistry Center—Institute of Molecular Sciences (CQC-IMS), University of Coimbra, 3004-535 Coimbra, Portugal

[5]CNC—Center for Neuroscience and Cell Biology, 3004-504 Coimbra, Portugal

[6]Institute for Interdisciplinary Research, University of Coimbra, 3030-789 Coimbra, Portugal

Table S1
Reactions, rate expressions, and parameter values considered in the kinetic model.

	Reactions	Reaction rates (v) and parameters	References
**1'**	H_2O_{2per} + $sPSH_{per}$ → $sPSOH_{per}$	K_1 = 100 $M^{-1}.s^{-1}$	See M.M.
**1**	H_2O_{2per} + PSH_{per} → $PSOH_{per}$	K_1' = 2.7 $M^{-1}.s^{-1}$	[72]
**2**	(s)$PSOH_{per}$ + H_2O_{2per} → (s)PSO_2H_{per}	K_2 = 100 $M^{-1}.s^{-1}$	[66,88]
**3**	(s)$PSOH_{per}$ + GSH_{per} → (s)$PSSG_{per}$	K_3 = 10 $M^{-1}.s^{-1}$	See M.M.
**4**	(s)PSH_{per} + $GSOH_{per}$ → (s)$PSSG_{per}$	K_4 = 10 $M^{-1}.s^{-1}$	See M.M.
**5,6**	(s)$PSSG_{per}$ + GSH_{per} ⇄ (s)PSH_{per} + $GSSG_{per}$	K_5 = K_6 = 1 $M^{-1}.s^{-1}$	[29]
**7,8**	$PSSP_{per}$ + GSH_{per} ⇄ PSH_{per} + $PSSG_{per}$	K_7 = K_8 = 1 $M^{-1}.s^{-1}$	[29]
**9**	PSH_{per} + $PSOH_{per}$ → $PSSP_{per}$	K_9= 10 $M^{-1}.s^{-1}$	See M.M.
**10**	→ H_2O_{2per}	V_{prod} = 3.6 × 10^{-4} $M.s^{-1}$	[82]; See M.M.
**11**	H_2O_{2per} + cat → cat	K_{cat}= 7.9 × 10^6 $M^{-1}.s^{-1}$	[86]
**12**	GSH_{per} + H_2O_{2per} → $GSOH_{per}$	$K_{sulfenic}$ = 0.87 $M^{-1}.s^{-1}$	[29]
**13**	GSH_{per} + $GSOH_{per}$ → $GSSG_{per}$	$K_{cond.}$ = 10 $M^{-1}.s^{-1}$	See M.M.
**14**	→ PSH_{per}	V_{imp} = 2.27 × 10^{-8} $M.s^{-1}$	See M.M.
**15**	→ $sPSH_{per}$	V_{imp} = 4.94 × 10^{-10} $M.s^{-1}$	See M.M.
**16**	(s)PSH_{per}/SOH_{per}/SO_2H_{per}/SG_{per}; $PSSP_{per}$ → deg	K_{degr}= 4.94 × 10^{-6} s^{-1}	See M.M.
**17**	GSH_{cyt} + $GSOH_{cyt}$ → $GSSG_{cyt}$	$K_{cond.}$ = 10 $M^{-1}.s^{-1}$	See M.M.
**18**	$GSSG_{cyt}$ + NADPH → 2 GSH_{cyt} + NADP	$V_{max(rGR)}$ = 1.78 x 10^{-4} $M.s^{-1}$<br>$K_{m(NADPH)}$ = 7.9 x 10^{-6} M<br>$K_{m(GSSG)}$= 5.67 x 10^{-5} M	[91]<br>[92]
**19**	H_2O_{2cyt} + GPX1red → GPX1ox	K_{ox} = 27 × 10^7 $M^{-1}.s^{-1}$	[90]
**20**	GPX1ox + GSH_{cyt} → GPX1-SG	K_{glut}= 4 × 10^4 $M^{-1}.s^{-1}$	[90]
**21**	GPX1-SG + GSH_{cyt} → GPX1red + $GSSG_{cyt}$	K_{red}=1 × 10^7 $M^{-1}.s^{-1}$	[91]
**22**	PrdxSH + H_2O_{2cyt}→PrdxSOH	$K_{sulfenic}$=1 × 10^8 $M^{-1}.s^{-1}$	See M.M.
**23**	PrdxSOH → PrdxSS	$K_{resolut}$ = 8.7 $M^{-1}.s^{-1}$	See M.M.
**24**	PrdxSS + TrxSH → PrdxSH + TrxSS	K_{red} = 2.3 × 10^6 $M^{-1}.s^{-1}$	See M.M.
**25**	NADPH + TrxSS → TrxSH + NADP*	$V_{max(TrxR)}$ = 5 × 10^{-5} $M.s^{-1}$<br>K_m =1.8 × 10^{-6} M	See M.M.
**26**	GSX_{per} ⇄ $GSX_{cyt;}$	K_{per} = 2.84 $M^{-1}.s^{-1}$	See M.M.
**27**	H_2O_{2per} ⇄ H_2O_{2cyt}	K_{per} = 284 $M^{-1}.s^{-1}$	[1]; See M.M.

*Although NADPH is a substrate in this reaction, it is not part of the rate equation (see M.M. for details). NADP was maintained in the rate equation to monitor NADPH consumption in this reaction.

Table S2
Species and initial concentrations used in the kinetic simulations.

Species	Initial concentration (M)	References
Catalase	5.6×10^{-4} (fixed)	[84,102]; See M.M.
H_2O_{2cyt}; H_2O_{2per}	0	
GSH_{cyt}	5×10^{-3}	[33]; See M.M.
GSH_{per}; $GSSG_{cyt}$; $GSSG_{per}$; $GSOH_{cyt}$; $GSOH_{per}$	0	
GPX1red	7.6×10^{-6}	See M.M.
GPX1ox; GPX1-SG	0	
NADPH	3.6×10^{-4} (fixed)	[93]
NADP	0	
$sPSH_{per}$	1×10^{-4}	See M.M.
$sPSOH_{per}$; $sPSO_2H_{per}$; $sPSSG_{per}$	0	
PSH_{per}	4.6×10^{-3}	[84,85]; See M.M.
$PSOH_{per}$; PSO_2H; $PSSP_{per;}$ $PSSG_{per}$	0	
PrdxSH	8.6×10^{-5}	See M.M.
PrdxSOH; PrdxSS	0	
TrxSH	6.3×10^{-5}	[94]
TrxSS	0	

Table S3

Concentrations of H_2O_2 and glutathione species (in M) at t= 48 hours (related to figure 6).

		**+ catalase + GSH/GSSG**	**+ catalase - GSH/GSSG**	**- catalase +GSH/GSSG**	**- catalase - GSH/GSSG**	**- GR/TrxR + catalase + GSH/GSSG**
	t=0	t= 48 h	t= 48 h	t= 48 h	t= 48 h	t= 48 h
H_2O_{2per}	0	7.6×10^{-8}	7.6×10^{-8}	1.3×10^{-6}	1.3×10^{-6}	8.1×10^{-8}
H_2O_{2cyt}	0	0.1×10^{-9}	0.1×10^{-9}	1.7×10^{-9}	1.7×10^{-9}	8.1×10^{-8}
GSH_{per}	0	4.8×10^{-3}	0	4.8×10^{-3}	0	1.0×10^{-8}
GSH_{cyt}	5×10^{-3}	4.8×10^{-3}	5.0×10^{-3}	4.8×10^{-3}	5.0×10^{-3}	1.6×10^{-9}
$GSOH_{per}$	0	0.3×10^{-9}	0	5.8×10^{-9}	0	1.6×10^{-10}
$GSOH_{cyt}$	0	0.2×10^{-9}	0	4.1×10^{-9}	0	1.6×10^{-10}
$GSSG_{per}$	0	5.9×10^{-9}	0	9.9×10^{-8}	0	2.3×10^{-3}
$GSSG_{cyt}$	0	5.3×10^{-9}	5.2×10^{-9}	8.9×10^{-8}	8.9×10^{-8}	2.3×10^{-3}
v_{NADPH} (M s^{-1})	0	9.0×10^{-7}	9.0×10^{-7}	1.5×10^{-5}	1.5×10^{-5}	0

Note that all metabolites shown reach quasi-steady-state concentrations in the first seconds/minutes of simulation. As a control, a simulation in which the cytosolic NADPH-dependent reductive system was omitted ("-GR/TrxR") was also performed. As expected, under these conditions almost all glutathione reaches the end of the simulation in the oxidized state. These conditions also result in the massive oxidation of sPper (99% of sPper is glutathionylated) and Pper (~2/3 as PSSPer and ~1/3 as PSSGper).

Supplementary Figure 1

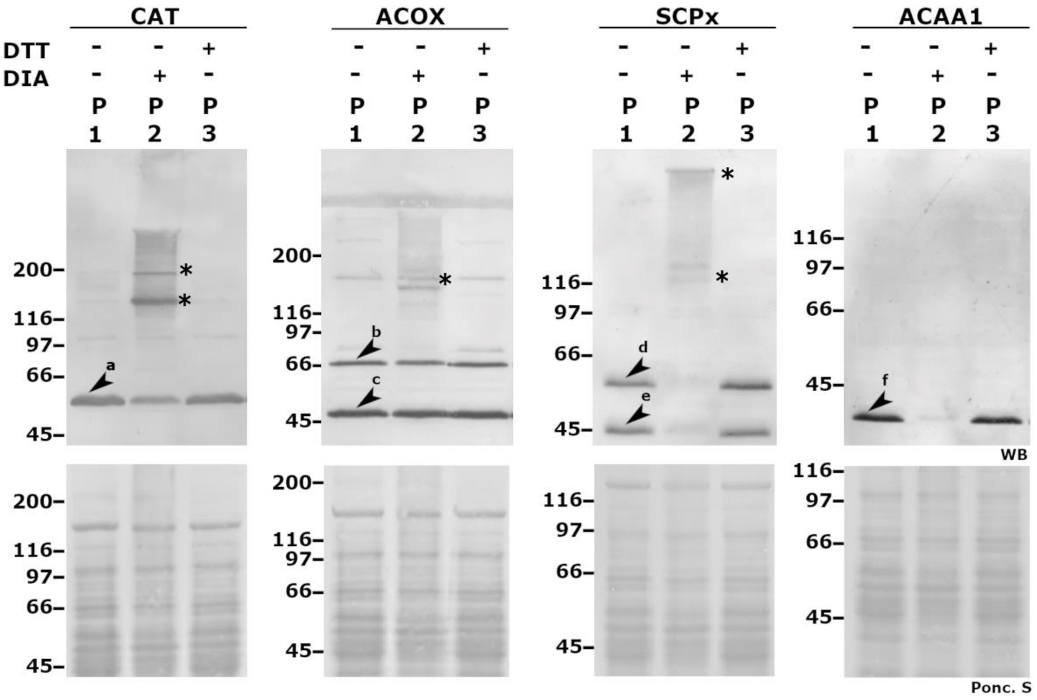

Figure S1 – Oxidation of endogenous rat liver peroxisomal proteins with diamide. PNSs in import buffer were incubated for 15 min at 37 °C in the absence (lanes 1) or presence of either 1 mM diamide (DIA; lanes 2) or 10 mM dithiothreitol (DTT; lanes 3). After adding NEM (30 mM final concentration), the PNSs were centrifuged and the organelle fraction was subjected to non-reducing SDS-PAGE/western-blotting (WB) using antibodies directed to catalase ("CAT"; arrow a), acyl-CoA oxidase 1 ("ACOX"; arrows b and c), sterol carrier protein x ("SCPx"; arrows d and e), and peroxisomal 3-ketoacyl-CoA thiolase 1a ("ACAA1"; arrow f). Oxidation products are marked with asterisks. Note that although monomeric ACAA1 is not detectable upon diamide treatment, no oxidation products are visible, possibly because they are too heterogeneous. The Ponceau S-stained membranes ("Ponc. S") are also shown; numbers to the left indicate the molecular weight markers (in kDa).

Supplementary Figure 2

	[PSHper]	[PSOHper]	[PSSGper]	[PSO2Hper]	[PSSPper]	[sPSHper]	[sPSOHper]	[sPSSGper]	[sPSO2Hper]	[H2O2per]	[GSHper]	[GSSGper]	[GSOHper]	[H2O2cyt]	[GSHcyt]	[GSSGcyt]	[GSOHcyt]	[GPX1o]	[GPX1r]	[GPX1GS]	[PrdxSH]	[PrdxSOH]	[PrdxSS]	[TrxSH]	[TrxSS]
H2O2 production	0	1.	1.	2.	1.	0	1.	1.	2.	1	0	1.	1.	1.	0	1.	1.	1.	0	1.	0	1.	1.	0	1.
Catalaseper reaction	0	−0.93	−0.93	−1.9	−0.93	0	−0.93	−0.93	−1.9	−0.93	0	−0.93	−0.93	−0.93	0	−0.93	−0.93	−0.93	0	−0.93	0	−0.93	−0.93	0	−0.95
kpS_H2O2	0	−0.06	−0.036	−0.12	−0.044	0	−0.06	−0.059	−0.12	−0.06	0	0.83	−0.06	0.94	0	0.93	−0.06	0.94	0	0.94	0	0.94	0.94	0	0.95
PSHper import	1.	0.51	1.	0.51	1.8	0	0	0	0	0	0	0.06	−0.05	0	0	0	−0.05	0	0	0	0	0	0	0	0
PSHper degradation	−0.99	−0.51	−0.99	−0.51	−1.8	0	0	0	0	0	0	−0.059	0.05	0	0	0	0.05	0	0	0	0	0	0	0	0
PrdxSH sulfenylation	0	0	−0.024	0	−0.016	0	0	0	0	0	0	−0.87	0	−0.97	0	−0.97	0	−0.97	0	−0.97	0	0.019	0.019	0	0.02
sPSHper import	0	0	0	0	0	1	1.	1.	1.	0	0	0.047	0	0	0	0	0	0	0	0	0	0	0	0	0
sPSHper degradation	0	0	0	0	0	−0.99	−0.99	−0.99	−0.99	0	0	−0.047	0	0	0	0	0	0	0	0	0	0	0	0	0
PSHper sulfenylation	0	1.	0.96	1.	0.97	0	0	0	0	0	0	0.059	0	0	0	0	0	0	0	0	0	0	0	0	0
GPX1_R1	0	0	0.024	0	0.016	0	0	0	0	0	0	0.87	0	−0.018	0	0.98	0	0.98	0	0.98	0	−0.018	−0.018	0	−0.019
sPSHper sulfenylation	0	0	0	0	0	0	1.	1.	1.	0	0	0.047	0	0	0	0	0	0	0	0	0	0	0	0	0
GSHper sulfenylation	0	0	0.016	0	0.011	0	0	0	0	0	0	0	1.	0	0	0	1.	0	0	0	0	0	0	0	0
sPSOHper glutathionylation	0	0	0	0	0	0	−0.99	0	−0.99	0	0	0	0	0	0	0	0	0	0	0	0	0	0	0	0
(GRcyt).KMA	0	0	0.024	0	0.016	0	0	0	0	0	0	0.9	0	0	0	1	0	0	0	0	0	0	0	0	0
(GRcyt).Vmax	0	0	−0.024	0	−0.016	0	0	0	0	0	0	−0.89	0	0	0	−0.99	0	0	0	0	0	0	0	0	0
PSSGper GSH TDE	0	0	−0.99	0	−0.66	0	0	0	0	0	0	0	0	0	0	0	0	0	0	0	0	0	0	0	0
GSOHcyt GSHcyt condensation	0	0	−0.01	0	0	0	0	0	0	0	0	0	−0.63	0	0	0	−0.92	0	0	0	0	0	0	0	0
PSOHper glutathionylation	0	−0.51	0	−0.51	−0.17	0	0	0	0	0	0	0	0	0	0	0	0	0	0	0	0	0	0	0	0
PSHper + PSOHper condensation	0	−0.49	0	−0.49	0.17	0	0	0	0	0	0	0	0	0	0	0	0	0	0	0	0	0	0	0	0
(TrxR).V	0	0	0	0	0	0	0	0	0	0	0	0	0	0	0	0	0	0	0	0	0	0	0	0	−1.
(TrxR).Km	0	0	0	0	0	0	0	0	0	0	0	0	0	0	0	0	0	0	0	0	0	0	0	0	1
sPSOHper sulfinylation	0	0	0	0	0	0	0	0	1.	0	0	0	0	0	0	0	0	0	0	0	0	0	0	0	0
PSHper sulfinylation	0	0	0	1.	0	0	0	0	0	0	0	0	0	0	0	0	0	0	0	0	0	0	0	0	0
PrdxSOH resolution	0	0	0	0	0	0	0	0	0	0	0	0	0	0	0	0	0	0	0	0	0	−0.99	0	0	0
sPSSGper GSH TDE	0	0	0	0	0	0	0	−0.99	0	0	0	0	0	0	0	0	0	0	0	0	0	0	0	0	0
PrdxSS reduction	0	0	0	0	0	0	0	0	0	0	0	0	0	0	0	0	0	0	0	0	0	0	−0.99	0	0
sPSO2Hper degradation	0	0	0	0	0	0	0	0	−0.99	0	0	0	0	0	0	0	0	0	0	0	0	0	0	0	0
PSO2Hper degradation	0	0	0	−0.99	0	0	0	0	0	0	0	0	0	0	0	0	0	0	0	0	0	0	0	0	0
GPX1_R2	0	0	0	0	0	0	0	0	0	0	0	0	0	0	0	0	0	−0.99	0	0	0	0	0	0	0
GPX1_R3	0	0	0	0	0	0	0	0	0	0	0	0	0	0	0	0	0	0	0	−0.99	0	0	0	0	0
PSSPper GSH TDE	0	0	0	0	−0.99	0	0	0	0	0	0	0	0	0	0	0	0	0	0	0	0	0	0	0	0
PSSPper GSH TDE reverse	0	0	0	0	0.67	0	0	0	0	0	0	0	0	0	0	0	0	0	0	0	0	0	0	0	0
kpS_GS	0	0	0	0	0	0	0	0	0	0	0	−0.1	−0.26	0	0	0	0.03	0	0	0	0	0	0	0	0
PSHper GSOH condensation	0	0	0.015	0	0.01	0	0	0	0	0	0	0	−0.05	0	0	0	−0.05	0	0	0	0	0	0	0	0
GSOHper GSHper condensation	0	0	0	0	0	0	0	0	0	0	0	0	−0.052	0	0	0	−0.052	0	0	0	0	0	0	0	0
PSSGper GSH TDE reverse	0	0	0.027	0	0.018	0	0	0	0	0	0	0	0	0	0	0	0	0	0	0	0	0	0	0	0
sPSSGper degradation	0	0	0	0	0	0	0	0	0	0	0	0	0	0	0	0	0	0	0	0	0	0	0	0	0
PSSPper degradation	0	0	0	0	0	0	0	0	0	0	0	0	0	0	0	0	0	0	0	0	0	0	0	0	0
PSSGper degradation	0	0	0	0	0	0	0	0	0	0	0	0	0	0	0	0	0	0	0	0	0	0	0	0	0
sPSHper GSOH condensation	0	0	0	0	0	0	0	0	0	0	0	0	0	0	0	0	0	0	0	0	0	0	0	0	0
sPSOHper degradation	0	0	0	0	0	0	0	0	0	0	0	0	0	0	0	0	0	0	0	0	0	0	0	0	0
sPSSGper GSH TDE reverse	0	0	0	0	0	0	0	0	0	0	0	0	0	0	0	0	0	0	0	0	0	0	0	0	0
PSOHper degradation	0	0	0	0	0	0	0	0	0	0	0	0	0	0	0	0	0	0	0	0	0	0	0	0	0
(GRcyt).KMB	0	0	0	0	0	0	0	0	0	0	0	0	0	0	0	0	0	0	0	0	0	0	0	0	0

Figure S2 – Sensitivity analysis of the kinetic model. Scaled sensitivities of the steady state concentrations to the kinetic parameters of the indicated reactions for the reference conditions. A scaled sensitivity of *x* of concentration *C* to parameter *p* means approximately that a 1% increase in *p* will cause a *x*% change in *C*. Positive and negative sensitivities are indicated in green and red, respectively, the color saturation being proportional to the absolute value. Sensitivities lower than 0.01 in absolute value were rounded to 0 for ease of visualization. The kinetic parameters are sorted by overall influence, expressed as the sum of the absolute values of the scaled sensitivities of all the concentrations.

Supplementary Figure 3

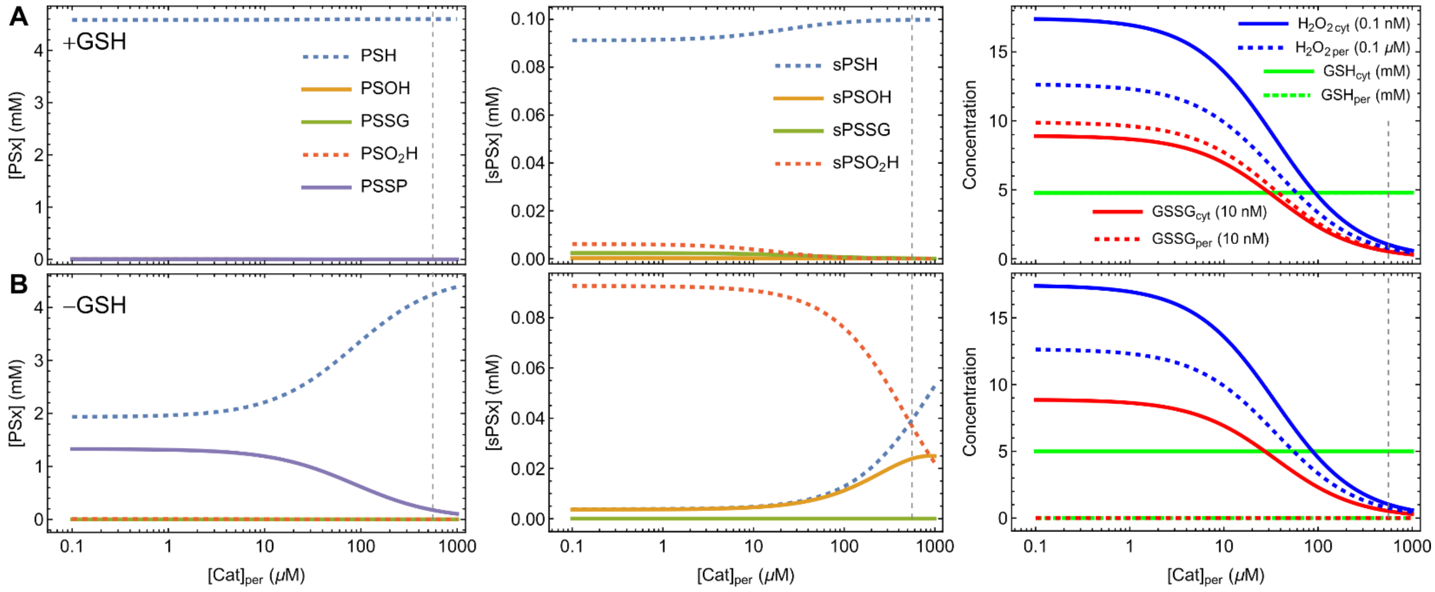

Figure S3 – Effect of catalase concentration on the redox state of Pper and sPper and concentrations of H_2O_2, GSH and GSSG. Simulations were performed with a peroxisome that has a membrane permeable (row A) or impermeable (row B) to glutathione. The dashed vertical line indicates the catalase concentration used in the reference condition. PSH, PSOH, PSO_2H, PSSG, PSSP represent the pools of total peroxisomal protein in the reduced, sulfenic, sulfinic, glutathionylated and dimeric state, respectively; sPSH, sPSOH, $sPSO_2H$, sPSSG represent the pools of hydrogen peroxide-sensitive peroxisomal protein in the reduced, sulfenic, sulfinic, and glutathionylated state, respectively.

Supplementary Figure 4

$$\frac{d(\mathrm{[H2O2per]}\cdot V_{\mathrm{peroxisome}})}{dt} = +V_{\mathrm{peroxisome}}\cdot(0.000359) - V_{\mathrm{peroxisome}}\cdot(7.9e{+}06\cdot\mathrm{[H2O2per]}\cdot\mathrm{[Catalaseper]}) - ((\mathrm{kpS\_H2O2}\cdot\mathrm{[H2O2per]} - \mathrm{kpS\_H2O2}\cdot\mathrm{[H2O2cyt]})) - V_{\mathrm{peroxisome}}\cdot(0.87\cdot\mathrm{[GSHper]}\cdot\mathrm{[H2O2per]}) - V_{\mathrm{peroxisome}}\cdot(2.7\cdot\mathrm{[PSHper]}\cdot\mathrm{[H2O2per]}) - V_{\mathrm{peroxisome}}\cdot(100\cdot\mathrm{[PSOHper]}\cdot\mathrm{[H2O2per]}) - V_{\mathrm{peroxisome}}\cdot(100\cdot\mathrm{[sPSHper]}\cdot\mathrm{[H2O2per]}) - V_{\mathrm{peroxisome}}\cdot(100\cdot\mathrm{[sPSOHper]}\cdot\mathrm{[H2O2per]})$$

$$\frac{d(\mathrm{[GSHper]}\cdot V_{\mathrm{peroxisome}})}{dt} = -V_{\mathrm{peroxisome}}\cdot(10\cdot\mathrm{[sPSOHper]}\cdot\mathrm{[GSHper]}) - V_{\mathrm{peroxisome}}\cdot((1\cdot\mathrm{[sPSSGper]}\cdot\mathrm{[GSHper]} - 1\cdot\mathrm{[sPSHper]}\cdot\mathrm{[GSSGper]})) - ((\mathrm{kpS\_GS}\cdot\mathrm{[GSHper]} - \mathrm{kpS\_GS}\cdot\mathrm{[GSHcyt]})) - V_{\mathrm{peroxisome}}\cdot(0.87\cdot\mathrm{[GSHper]}\cdot\mathrm{[H2O2per]}) - V_{\mathrm{peroxisome}}\cdot((1\cdot\mathrm{[PSSPper]}\cdot\mathrm{[GSHper]} - 1\cdot\mathrm{[PSHper]}\cdot\mathrm{[PSSGper]})) - V_{\mathrm{peroxisome}}\cdot(10\cdot\mathrm{[GSOHper]}\cdot\mathrm{[GSHper]}) - V_{\mathrm{peroxisome}}\cdot(10\cdot\mathrm{[PSOHper]}\cdot\mathrm{[GSHper]}) - V_{\mathrm{peroxisome}}\cdot((1\cdot\mathrm{[PSSGper]}\cdot\mathrm{[GSHper]} - 1\cdot\mathrm{[PSHper]}\cdot\mathrm{[GSSGper]}))$$

$$\frac{d(\mathrm{[GSSGper]}\cdot V_{\mathrm{peroxisome}})}{dt} = +V_{\mathrm{peroxisome}}\cdot((1\cdot\mathrm{[sPSSGper]}\cdot\mathrm{[GSHper]} - 1\cdot\mathrm{[sPSHper]}\cdot\mathrm{[GSSGper]})) - ((\mathrm{kpS\_GS}\cdot\mathrm{[GSSGper]} - \mathrm{kpS\_GS}\cdot\mathrm{[GSSGcyt]})) + V_{\mathrm{peroxisome}}\cdot(10\cdot\mathrm{[GSOHper]}\cdot\mathrm{[GSHper]}) + V_{\mathrm{peroxisome}}\cdot((1\cdot\mathrm{[PSSGper]}\cdot\mathrm{[GSHper]} - 1\cdot\mathrm{[PSHper]}\cdot\mathrm{[GSSGper]}))$$

$$\frac{d(\mathrm{[GSOHper]}\cdot V_{\mathrm{peroxisome}})}{dt} = -V_{\mathrm{peroxisome}}\cdot(10\cdot\mathrm{[sPSHper]}\cdot\mathrm{[GSOHper]}) - ((\mathrm{kpS\_GS}\cdot\mathrm{[GSOHper]} - \mathrm{kpS\_GS}\cdot\mathrm{[GSOHcyt]})) + V_{\mathrm{peroxisome}}\cdot(0.87\cdot\mathrm{[GSHper]}\cdot\mathrm{[H2O2per]}) - V_{\mathrm{peroxisome}}\cdot(10\cdot\mathrm{[GSOHper]}\cdot\mathrm{[GSHper]}) - V_{\mathrm{peroxisome}}\cdot(10\cdot\mathrm{[PSHper]}\cdot\mathrm{[GSOHper]})$$

$$\frac{d(\mathrm{[GPX1o]}\cdot V_{\mathrm{cytosol}})}{dt} = +V_{\mathrm{cytosol}}\cdot(2.1e{+}07\cdot\mathrm{[GPX1r]}\cdot\mathrm{[H2O2cyt]}) - V_{\mathrm{cytosol}}\cdot(40000\cdot\mathrm{[GPX1o]}\cdot\mathrm{[GSHcyt]})$$

$$\frac{d(\mathrm{[GPX1r]}\cdot V_{\mathrm{cytosol}})}{dt} = -V_{\mathrm{cytosol}}\cdot(2.1e{+}07\cdot\mathrm{[GPX1r]}\cdot\mathrm{[H2O2cyt]}) + V_{\mathrm{cytosol}}\cdot(1e{+}07\cdot\mathrm{[GPX1GS]}\cdot\mathrm{[GSHcyt]})$$

$$\frac{d(\mathrm{[GPX1GS]}\cdot V_{\mathrm{cytosol}})}{dt} = +V_{\mathrm{cytosol}}\cdot(40000\cdot\mathrm{[GPX1o]}\cdot\mathrm{[GSHcyt]}) - V_{\mathrm{cytosol}}\cdot(1e{+}07\cdot\mathrm{[GPX1GS]}\cdot\mathrm{[GSHcyt]})$$

$$\frac{d(\mathrm{[sPSHper]}\cdot V_{\mathrm{peroxisome}})}{dt} = +V_{\mathrm{peroxisome}}\cdot((1\cdot\mathrm{[sPSSGper]}\cdot\mathrm{[GSHper]} - 1\cdot\mathrm{[sPSHper]}\cdot\mathrm{[GSSGper]})) - V_{\mathrm{peroxisome}}\cdot(10\cdot\mathrm{[sPSHper]}\cdot\mathrm{[GSOHper]}) - V_{\mathrm{peroxisome}}\cdot(4.93694e{-}06\cdot\mathrm{[sPSHper]}) + V_{\mathrm{peroxisome}}\cdot(4.937e{-}10) - V_{\mathrm{peroxisome}}\cdot(100\cdot\mathrm{[sPSHper]}\cdot\mathrm{[H2O2per]})$$

$$\frac{d(\mathrm{[sPSOHper]}\cdot V_{\mathrm{peroxisome}})}{dt} = -V_{\mathrm{peroxisome}}\cdot(10\cdot\mathrm{[sPSOHper]}\cdot\mathrm{[GSHper]}) - V_{\mathrm{peroxisome}}\cdot(4.93694e{-}06\cdot\mathrm{[sPSOHper]}) + V_{\mathrm{peroxisome}}\cdot(100\cdot\mathrm{[sPSHper]}\cdot\mathrm{[H2O2per]}) - V_{\mathrm{peroxisome}}\cdot(100\cdot\mathrm{[sPSOHper]}\cdot\mathrm{[H2O2per]})$$

$$\frac{d(\mathrm{[sPSO2Hper]}\cdot V_{\mathrm{peroxisome}})}{dt} = -V_{\mathrm{peroxisome}}\cdot(4.93694e{-}06\cdot\mathrm{[sPSO2Hper]}) + V_{\mathrm{peroxisome}}\cdot(100\cdot\mathrm{[sPSOHper]}\cdot\mathrm{[H2O2per]})$$

$$\frac{d(\mathrm{[sPSSGper]}\cdot V_{\mathrm{peroxisome}})}{dt} = +V_{\mathrm{peroxisome}}\cdot(10\cdot\mathrm{[sPSOHper]}\cdot\mathrm{[GSHper]}) - V_{\mathrm{peroxisome}}\cdot((1\cdot\mathrm{[sPSSGper]}\cdot\mathrm{[GSHper]} - 1\cdot\mathrm{[sPSHper]}\cdot\mathrm{[GSSGper]})) + V_{\mathrm{peroxisome}}\cdot(10\cdot\mathrm{[sPSHper]}\cdot\mathrm{[GSOHper]}) - V_{\mathrm{peroxisome}}\cdot(4.93694e{-}06\cdot\mathrm{[sPSSGper]})$$

$$\frac{d(\mathrm{[GSOHcyt]}\cdot V_{\mathrm{cytosol}})}{dt} = -V_{\mathrm{cytosol}}\cdot(10\cdot\mathrm{[GSOHcyt]}\cdot\mathrm{[GSHcyt]}) + ((\mathrm{kpS\_GS}\cdot\mathrm{[GSOHper]} - \mathrm{kpS\_GS}\cdot\mathrm{[GSOHcyt]}))$$

$$\frac{d(\mathrm{[PSHper]}\cdot V_{\mathrm{peroxisome}})}{dt} = -V_{\mathrm{peroxisome}}\cdot(10\cdot\mathrm{[PSHper]}\cdot\mathrm{[PSOHper]}) + V_{\mathrm{peroxisome}}\cdot((1\cdot\mathrm{[PSSPper]}\cdot\mathrm{[GSHper]} - 1\cdot\mathrm{[PSHper]}\cdot\mathrm{[PSSGper]})) - V_{\mathrm{peroxisome}}\cdot(4.93694e{-}06\cdot\mathrm{[PSHper]}) + V_{\mathrm{peroxisome}}\cdot(2.271e{-}08) - V_{\mathrm{peroxisome}}\cdot(2.7\cdot\mathrm{[PSHper]}\cdot\mathrm{[H2O2per]}) + V_{\mathrm{peroxisome}}\cdot((1\cdot\mathrm{[PSSGper]}\cdot\mathrm{[GSHper]} - 1\cdot\mathrm{[PSHper]}\cdot\mathrm{[GSSGper]})) - V_{\mathrm{peroxisome}}\cdot(10\cdot\mathrm{[PSHper]}\cdot\mathrm{[GSOHper]})$$

$$\frac{d(\mathrm{[PSOHper]}\cdot V_{\mathrm{peroxisome}})}{dt} = -V_{\mathrm{peroxisome}}\cdot(10\cdot\mathrm{[PSHper]}\cdot\mathrm{[PSOHper]}) - V_{\mathrm{peroxisome}}\cdot(4.93694e{-}06\cdot\mathrm{[PSOHper]}) + V_{\mathrm{peroxisome}}\cdot(2.7\cdot\mathrm{[PSHper]}\cdot\mathrm{[H2O2per]}) - V_{\mathrm{peroxisome}}\cdot(100\cdot\mathrm{[PSOHper]}\cdot\mathrm{[H2O2per]}) - V_{\mathrm{peroxisome}}\cdot(10\cdot\mathrm{[PSOHper]}\cdot\mathrm{[GSHper]})$$

$$\frac{d(\mathrm{[PSSPper]}\cdot V_{\mathrm{peroxisome}})}{dt} = +V_{\mathrm{peroxisome}}\cdot(10\cdot\mathrm{[PSHper]}\cdot\mathrm{[PSOHper]}) - V_{\mathrm{peroxisome}}\cdot((1\cdot\mathrm{[PSSPper]}\cdot\mathrm{[GSHper]} - 1\cdot\mathrm{[PSHper]}\cdot\mathrm{[PSSGper]})) - V_{\mathrm{peroxisome}}\cdot(4.93694e{-}06\cdot\mathrm{[PSSPper]})$$

$$\frac{d(\mathrm{[PSSGper]}\cdot V_{\mathrm{peroxisome}})}{dt} = +V_{\mathrm{peroxisome}}\cdot((1\cdot\mathrm{[PSSPper]}\cdot\mathrm{[GSHper]} - 1\cdot\mathrm{[PSHper]}\cdot\mathrm{[PSSGper]})) - V_{\mathrm{peroxisome}}\cdot(4.93694e{-}06\cdot\mathrm{[PSSGper]}) + V_{\mathrm{peroxisome}}\cdot(10\cdot\mathrm{[PSOHper]}\cdot\mathrm{[GSHper]}) - V_{\mathrm{peroxisome}}\cdot((1\cdot\mathrm{[PSSGper]}\cdot\mathrm{[GSHper]} - 1\cdot\mathrm{[PSHper]}\cdot\mathrm{[GSSGper]})) + V_{\mathrm{peroxisome}}\cdot(10\cdot\mathrm{[PSHper]}\cdot\mathrm{[GSOHper]})$$

$$\frac{d(\mathrm{[PrdxSH]}\cdot V_{\mathrm{cytosol}})}{dt} = -V_{\mathrm{cytosol}}\cdot(1e{+}08\cdot\mathrm{[PrdxSH]}\cdot\mathrm{[H2O2cyt]}) + V_{\mathrm{cytosol}}\cdot(2.3e{+}06\cdot\mathrm{[PrdxSS]}\cdot\mathrm{[TrxSH]})$$

$$\frac{d(\mathrm{[PrdxSOH]}\cdot V_{\mathrm{cytosol}})}{dt} = +V_{\mathrm{cytosol}}\cdot(1e{+}08\cdot\mathrm{[PrdxSH]}\cdot\mathrm{[H2O2cyt]}) - V_{\mathrm{cytosol}}\cdot(8.7\cdot\mathrm{[PrdxSOH]})$$

$$\frac{d(\mathrm{[PrdxSS]}\cdot V_{\mathrm{cytosol}})}{dt} = +V_{\mathrm{cytosol}}\cdot(8.7\cdot\mathrm{[PrdxSOH]}) - V_{\mathrm{cytosol}}\cdot(2.3e{+}06\cdot\mathrm{[PrdxSS]}\cdot\mathrm{[TrxSH]})$$

$$\frac{d(\mathrm{[TrxSH]}\cdot V_{\mathrm{cytosol}})}{dt} = -V_{\mathrm{cytosol}}\cdot(2.3e{+}06\cdot\mathrm{[PrdxSS]}\cdot\mathrm{[TrxSH]}) + V_{\mathrm{cytosol}}\cdot\left(\frac{5e{-}05\cdot\mathrm{[TrxSS]}}{1.8e{-}06 + \mathrm{[TrxSS]}}\right)$$

$$\frac{d(\mathrm{[TrxSS]}\cdot V_{\mathrm{cytosol}})}{dt} = +V_{\mathrm{cytosol}}\cdot(2.3e{+}06\cdot\mathrm{[PrdxSS]}\cdot\mathrm{[TrxSH]}) - V_{\mathrm{cytosol}}\cdot\left(\frac{5e{-}05\cdot\mathrm{[TrxSS]}}{1.8e{-}06 + \mathrm{[TrxSS]}}\right)$$

$$\frac{d(\mathrm{[PSO2Hper]}\cdot V_{\mathrm{peroxisome}})}{dt} = +V_{\mathrm{peroxisome}}\cdot(100\cdot\mathrm{[PSOHper]}\cdot\mathrm{[H2O2per]}) - V_{\mathrm{peroxisome}}\cdot(4.93694e{-}06\cdot\mathrm{[PSO2Hper]})$$

$$\frac{d(\mathrm{[H2O2cyt]}\cdot V_{\mathrm{cytosol}})}{dt} = +((\mathrm{kpS\_H2O2}\cdot\mathrm{[H2O2per]} - \mathrm{kpS\_H2O2}\cdot\mathrm{[H2O2cyt]})) - V_{\mathrm{cytosol}}\cdot(1e{+}08\cdot\mathrm{[PrdxSH]}\cdot\mathrm{[H2O2cyt]}) - V_{\mathrm{cytosol}}\cdot(2.1e{+}07\cdot\mathrm{[GPX1r]}\cdot\mathrm{[H2O2cyt]})$$

$$\frac{d(\mathrm{[GSHcyt]}\cdot V_{\mathrm{cytosol}})}{dt} = -V_{\mathrm{cytosol}}\cdot(10\cdot\mathrm{[GSOHcyt]}\cdot\mathrm{[GSHcyt]}) + ((\mathrm{kpS\_GS}\cdot\mathrm{[GSHper]} - \mathrm{kpS\_GS}\cdot\mathrm{[GSHcyt]})) - V_{\mathrm{cytosol}}\cdot(40000\cdot\mathrm{[GPX1o]}\cdot\mathrm{[GSHcyt]}) - V_{\mathrm{cytosol}}\cdot(1e{+}07\cdot\mathrm{[GPX1GS]}\cdot\mathrm{[GSHcyt]}) + 2\cdot V_{\mathrm{cytosol}}\cdot\left(\frac{0.000178\cdot\mathrm{[GSSGcyt]}\cdot\mathrm{[NADPH]}}{5.67e{-}05\cdot\mathrm{[NADPH]} + 7.9e{-}06\cdot\mathrm{[GSSGcyt]} + \mathrm{[GSSGcyt]}\cdot\mathrm{[NADPH]}}\right)$$

$$\frac{d(\mathrm{[GSSGcyt]}\cdot V_{\mathrm{cytosol}})}{dt} = +V_{\mathrm{cytosol}}\cdot(10\cdot\mathrm{[GSOHcyt]}\cdot\mathrm{[GSHcyt]}) + ((\mathrm{kpS\_GS}\cdot\mathrm{[GSSGper]} - \mathrm{kpS\_GS}\cdot\mathrm{[GSSGcyt]})) + V_{\mathrm{cytosol}}\cdot(1e{+}07\cdot\mathrm{[GPX1GS]}\cdot\mathrm{[GSHcyt]}) - V_{\mathrm{cytosol}}\cdot\left(\frac{0.000178\cdot\mathrm{[GSSGcyt]}\cdot\mathrm{[NADPH]}}{5.67e{-}05\cdot\mathrm{[NADPH]} + 7.9e{-}06\cdot\mathrm{[GSSGcyt]} + \mathrm{[GSSGcyt]}\cdot\mathrm{[NADPH]}}\right)$$

$$\mathrm{GStot} = \mathrm{GPX1GS.ParticleNumber} + \mathrm{GSHcyt.ParticleNumber} + \mathrm{GSHper.ParticleNumber} + \mathrm{GSOHcyt.ParticleNumber} + \mathrm{GSOHper.ParticleNumber} + 2\cdot\mathrm{GSSGcyt.ParticleNumber} + 2\cdot\mathrm{GSSGper.ParticleNumber} + \mathrm{PSSGper.ParticleNumber} + \mathrm{sPSSGper.ParticleNumber}$$

$$\mathrm{sPSpertot} = \mathrm{sPSHper.ParticleNumber} + \mathrm{sPSO2Hper.ParticleNumber} + \mathrm{sPSOHper.ParticleNumber} + \mathrm{sPSSGper.ParticleNumber}$$

$$\mathrm{PSpertot} = \mathrm{PSHper.ParticleNumber} + \mathrm{PSO2Hper.ParticleNumber} + \mathrm{sPSOHper.ParticleNumber} + \mathrm{sPSSGper.ParticleNumber} + 2\cdot\mathrm{PSSPper.ParticleNumber}$$

$$\mathrm{Prdxtot} = \mathrm{PrdxSH.ParticleNumber} + \mathrm{PrdxSOH.ParticleNumber} + \mathrm{PrdxSS.ParticleNumber}$$

$$\mathrm{GPxtot} = \mathrm{GPX1GS.ParticleNumber} + \mathrm{GPX1o.ParticleNumber} + \mathrm{GPX1r.ParticleNumber}$$

$$\mathrm{Trxtot} = \mathrm{TrxSH.ParticleNumber} + \mathrm{TrxSS.ParticleNumber}$$

$$\mathrm{fsPSox} = 1 - \frac{\mathrm{sPSHper.ParticleNumber}}{\mathrm{sPSpertot}}$$

$$\mathrm{fPSox} = 1 - \frac{\mathrm{PSHper.ParticleNumber}}{\mathrm{PSpertot}}$$

Figure S4 – Description of the cytosol-peroxisome redox relationship using an ordinary differential equation system.
